# Formulation and field performance of liquid phosphate-solubilizing bacterial biofertilizer for improved growth, yield, and nutrient content of potato

**DOI:** 10.3389/fmicb.2025.1734471

**Published:** 2025-12-03

**Authors:** Riya Bansal, Pratibha Vyas, Sandeep Sharma

**Affiliations:** 1 Department of Microbiology, College of Basic Sciences and Humanities, Punjab Agricultural University, Ludhiana, Punjab, India; 2 Department of Soil Science, College of Agriculture, Punjab Agricultural University, Ludhiana, Punjab, India

**Keywords:** *Bacillus licheniformis*, *Pseudomonas putida*, liquid formulation, phosphorus content, shelf life

## Abstract

In the present study, a liquid formulation(s) containing two phosphate-solubilizing *Bacillus licheniformis* PRPSB_10_ and *Pseudomonas putida* PRPSB_38_ has been developed and tested for its efficacy to improve growth, yield, and nutrient content of potato (*Solanum tuberosum* L.) under integrated nutrient management system. These two phosphate-solubilizing strains, isolated from potato rhizosphere showed high phosphate-solubilizing efficiency, with solubilization index of 6.1 and 5.8, respectively, on modified Pikovskaya agar. The strain PRPSB_10_ solubilized 659.9, 202.0, and 113.7 μg ml^−1^ of tricalcium phosphate, rock phosphate, and iron phosphate, respectively, while the strain PRPSB_38_ solubilized 457.4, 187.9, and 80.0 μg ml ^−1^ of the same substrates, respectively, after 5 days of incubation in NBRIP broth. Among the different cell protectants tested, 0.1% carboxymethyl cellulose (CMC) was found to be the best for *B. licheniformis* PRPSB_10_ while 5 mM trehalose was best suited for *P. putida* PRPSB_38_ maintaining the cell viability ~10^8^ CFU ml^−1^ up to 1 year under ambient storage conditions. Two-year field evaluations of the liquid formulation in potato variety *Kufri Pukhraj* with different P dosages revealed a significant improvement in plant growth, phosphorus content, tuber yield and soil fertility over the respective control treatment with an average increase of 16.2% in yield over control. Economic analysis further indicated mean net returns of ₹44,876 ha^−1^ and an improved benefit-cost ratio of 2.52 compared with the uninoculated control. The results indicated that the developed liquid PSB formulation exhibited excellent stability, effectively enhanced yield and phosphorus availability highlighting its potential to be used as a liquid biofertilizer for improving potato productivity and maintaining soil health.

## 1 Introduction

Potato (*Solanum tuberosum* L.), a member of the family Solanaceae, is a starchy and highly nutritious tuberous crop, commonly referred to as the “King of Vegetables.” Globally, it ranks third among food crops after wheat and rice, with Asia being the largest producer. India ranks second in production and third in terms of area under cultivation (Source: agropedia.iitk.ac.in). Major potato-producing states include Punjab, Uttar Pradesh, Bihar, Haryana, Assam, Madhya Pradesh, West Bengal, and Gujarat. In Punjab, potato is cultivated over approximately 117.06 thousand hectares, yielding an estimated 32.38 lakh tons during 2023–24, predominantly in Jalandhar, Hoshiarpur, Kapurthala, Ludhiana, Amritsar, Bathinda, and Fatehgarh Sahib districts (Anonymous, 2025).

Integrated nutrient management including the use of chemical fertilizers, organic inputs and biofertilizers is pivotal for achieving optimum potato productivity. While nitrogen and potassium uptake peaks during early growth (30–40 days post-emergence), phosphorus—the second most critical macronutrient after nitrogen, is indispensable throughout the crop cycle (Kaur et al., 2022). It regulates root and shoot development, energy transfer, starch synthesis and enzymatic activities, thereby enhancing tuber quality and dry matter accumulation (Vyas and Gulati, 2009; Kaur et al., 2022). As a structural component of nucleic acids, phospholipids, and ATP, phosphorus is integral to cell division, signal transduction, carbohydrate metabolism, photosynthesis, respiration, and overall plant development. Adequate P nutrition also accelerates early maturity, improves stress tolerance, and enhances produce quality.

Despite its importance, P availability is often limited (< 22.4 kg P ha^−1^) in nearly 50% of Punjab soils (Sharma et al., 2016), constraining crop productivity. Potato, being highly P-demanding, frequently requires fertilizer P applications exceeding 150 kg P_2_O_5_ ha^−1^ (Rosen et al., 2014). However, applied P is rapidly immobilized as insoluble Fe, Al, and Ca phosphates, resulting in fertilizer use efficiency rarely exceeding 30% (Ibrahim et al., 2022). Moreover, excessive P application poses environmental risks and relies on non-renewable phosphate rock, lead to be depleted within 50–100 years (Cordell et al., 2009).

Phosphate-solubilizing bacteria (PSB) are a group of beneficial bacteria which can hydrolyze organic and inorganic insoluble phosphate compounds into soluble P forms easily assimilated by plants (Li et al., 2025). These bacteria produce low molecular weight organic acids as well as phosphatases that release the soluble P which becomes available for plant absorption helping to reduce the P application in the form of inorganic fertilizers. Phosphate-solubilizing bacteria if supplemented with fertilizers can meet the high P demand of potato, thereby improving growth, yield and soil health.

Carrier-based inoculants have a limited shelf life, poor quality, high contamination and unpredictability in the field. A higher population of the desired microorganism, sufficient viability, and the ability to keep uncontaminated for an extended period, are all expected in high-quality biofertilizers. The advancements in inoculants technology are focused on enhancing quality, prolonging shelf life, and developing new formulations for usage in less favorable conditions. Additives/protectants including polymers (e.g. PVP, PEG, sodium alginate, gum arabic), adjuvants [e.g., carboxymethyl cellulose (CMC), xanthan gum, carrageenan], and surfactants (e.g., polysorbate 20, 40, 80) used in liquid inoculant formulations enhance the survival of bacterial inoculum in the field by protecting cells from abiotic stress and aiding their establishment in host plants (Allouzi et al., 2022). Trehalose (disaccharide) further improves microbial tolerance to desiccation, heat, and osmotic stress, while stabilizing enzymes and cell membranes (Kosar et al., 2019).

Effective PSB bioformulations require the use of compatible, well-characterized strains capable of synergistic activity in the rhizosphere. Looking into the importance of liquid formulations of plant growth-promoting and mineral-solubilizing bacteria, the present study was undertaken to develop a liquid phosphate-solubilizing bacterial (LPSB) formulation with enhanced shelf life for improved growth, yield and nutrient content of potato.

## 2 Materials and methods

### 2.1 Phosphate-solubilizing bacterial strains

Two efficient phosphate-solubilizing bacterial strains *Bacillus licheniformis* PRPSB_10_ (NCBI accession no. PV567726) and *Pseudomonas putida* PRPSB_38_ (NCBI accession no. PV567728) isolated from potato rhizosphere were obtained from the Department of Microbiology, Punjab Agricultural University, Ludhiana, India. The bacterial strains were purified using the quadrant streak method on nutrient agar and maintained on slants and stored in 30% (v/v) glycerol at temperature of −20 °C for further study.

### 2.2 Screening for phosphate solubilization

#### 2.2.1 Qualitative estimation of phosphate solubilization

Phosphate-solubilizing ability of the isolates was assessed on modified Pikovskayas agar containing 0.5% tricalcium phosphate (TCP) (Gupta et al., 1994). Fresh cultures were spot-inoculated and incubated at 28 °C for 5 days. Phosphate solubilization was indicated by yellow halos around the colonies (De Freitas et al., 1997), and the solubilization index was calculated from the ratio of total halo diameter (*z*) to colony diameter (*c*).


$$\begin{array}{l} Phosphate\;Solubilization\;Index\;\left(SI\right)=\frac{z}{c} \end{array}$$

#### 2.2.2 Quantitative estimation of phosphate solubilization

Quantitative phosphate solubilization was assessed in NBRIP broth (Nautiyal, 1999) containing 0.5% of tricalcium phosphate, iron phosphate, and rock phosphate (RP). A 500 μl (~10^9^ CFU ml^−1^) actively growing bacterial culture was inoculated into 50 ml broth and incubated at 28 °C on rotatory shaker at 120 rpm for 5 days. After incubation, cultures were centrifuged at 10,000 rpm for 10 min and soluble phosphate in the supernatant was estimated colorimetrically using Barton's reagent (Jackson, 1973). Absorbance was recorded at 430 nm and phosphate concentration (μg ml^−1^) was determined from a KH_2_PO_4_ standard curve. Uninoculated media containing phosphate substrates inoculated with 500 μl sterilized water and kept under similar conditions served as control.

### 2.3 Compatibility testing for PSB strains

Compatibility between *Bacillus licheniformis* PRPSB_10_ and *Pseudomonas putida* PRPSB_38_ was evaluated using the cross-streak method on nutrient agar plates (Sundaramoorthy et al., 2012; Vyas et al., 2025). Uniform growth at the intersection indicated compatibility, while inhibition indicated antagonism.

### 2.4 Testing pathogenicity of the PSB strains

Pathogenicity of PSB strains was evaluated on blood agar base supplemented with 5% defibrinated sheep blood and incubated at 37 °C for 24–48 h. Clear zones around colonies indicated hemolytic activity, classified as α-partial or β-complete hemolysis (Cruickshank et al., 1975).

### 2.5 Testing shelf-life of liquid formulation(s)

Liquid formulations of *B*. *licheniformis* PRPSB_10_ and *P*. *putida* PRPSB_38_ were prepared by growing the strains separately in nutrient broth (NB) supplemented with three different concentrations of each cell protectants/additives. A 500 μl (~10^9^ CFU ml^−1^) of actively growing bacterial culture was inoculated into 100 ml nutrient broth with and without amendments as given in Table 1.

**Table 1:** Treatment details for the development of liquid formulation.

**Treatment**	**Treatment details**
T1	Charcoal-based bacterial inoculant
T2	Unamended NB
T3	NB with 0.5% polyethylene glycol (PEG) 6000
T4	NB with 1.0% PEG 6000
T5	NB with 1.5% PEG 6000
T6	NB with 0.1% carboxymethyl cellulose (CMC)
T7	NB with 0.3% CMC
T8	NB with 0.5% CMC
T9	NB with 2.5 mM trehalose
T10	NB with 5 mM trehalose
T11	NB with 7.5 mM trehalose

For the preparation of charcoal-based bacterial inoculant, the bacterial strains grown in NB for 48 h were mixed separately with sterilized charcoal in the ratio 1:2 and packed in polyethylene packets and packets sealed properly using an electric sealer. The flasks and the charcoal-based inoculant were kept at room temperature for a period of 400 days and the viability of the bacterial strains was monitored at monthly intervals by serial plate dilution method and plating on nutrient agar. Colony count was recorded as colony-forming units per milliliter (CFU ml^−1^) to assess long-term stability.

### 2.6 Evaluation of PSB formulation(s) on growth, soil quality, nutrient acquisition and yield of potato under field conditions

#### 2.6.1 Preparation and application of liquid formulation of PSB (LPSBF)

**Figure 1:**
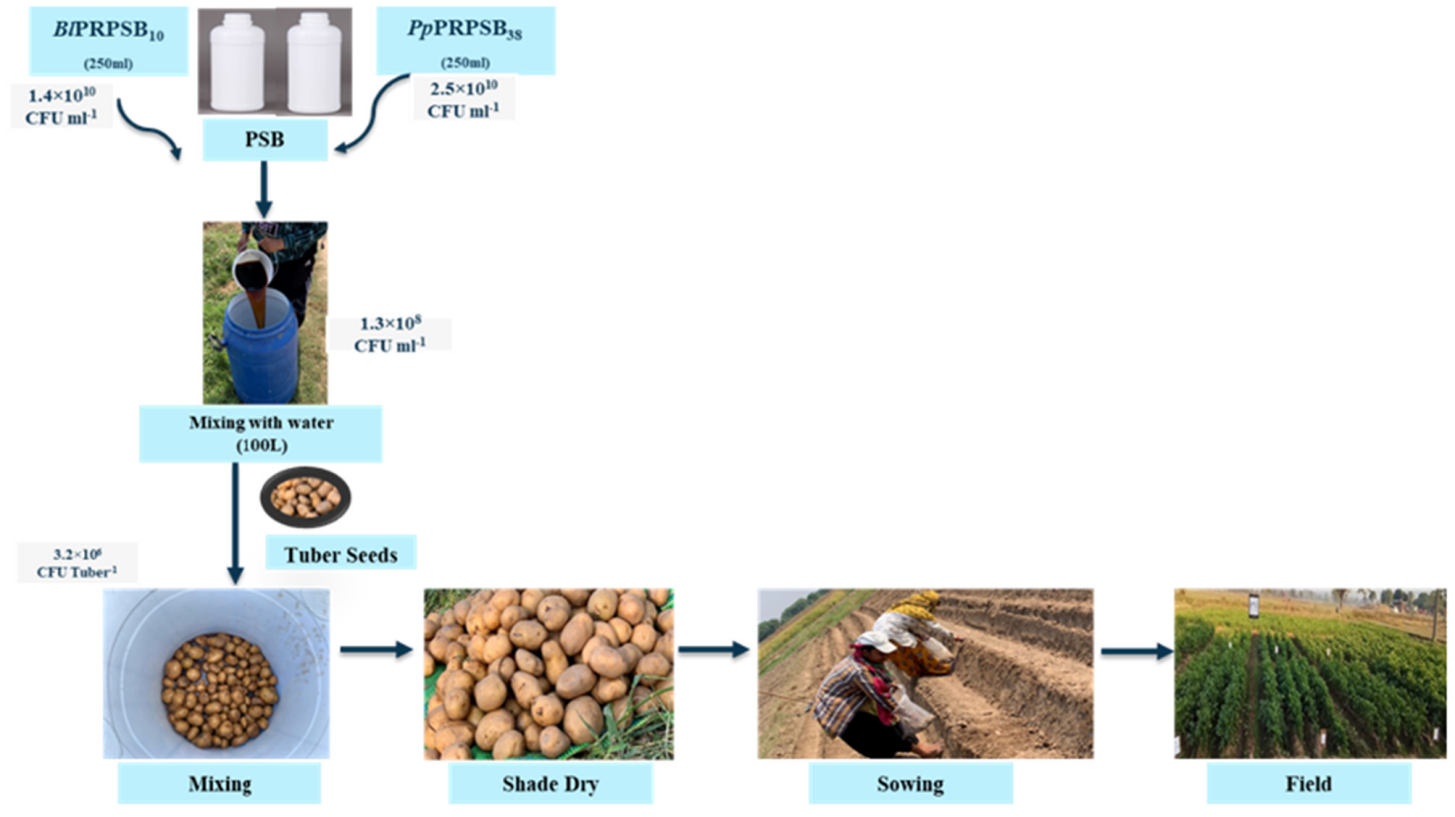
Application of liquid formulation of phosphate-solubilizing bacteria.

*Bacillus licheniformis* PRPSB_10_ and *Pseudomonas putida* PRPSB_38_ were cultured separately in 250 ml nutrient broth supplemented with 0.1% CMC and 5 mM trehalose, respectively, and incubated at 28 °C for 48 h (~10^10^ CFU ml^−1^). The formulations were diluted to 100 L of water to obtain 1.3 × 10^8^ CFU ml^−1^. Uniformly sized potato tubers (25–35 mm) were dipped in the suspension for 30 min to achieve 3.2 × 10^6^ CFU tuber^−1^, shade-dried and sown in the field. The treated tubers were then shade-dried and sown in the field as given in Figure 1.

#### 2.6.2 Field trials

Field trials were conducted using the potato variety *Kufri Pukhraj* at the Experimental Farms of the Department of Soil Science, PAU, Ludhiana, Punjab, India during the 2022–23 and 2023–24 during rabi seasons. The liquid PSB formulation (LPSBF) was tested in comparison to an uninoculated control and a charcoal-based consortium biofertilizer (CCB) developed and recommended by Punjab Agricultural University for potato crop (Anonymous, 2025). The experiments were carried out in a Randomized Block Design with 12 treatments and three replications as given in Table 2.

**Table 2:** Treatment details for field experiment.

**Treatment**	**Treatment details**
T1	NP_0_K + FYM
T2	NP_0_K + FYM + LPSBF
T3	NP_0_K + FYM + CCB
T4	NP_75_K + FYM
T5	NP_75_K + FYM + LPSBF
T6	NP_75_K + FYM + CCB
T7	NP_100_K + FYM
T8	NP_100_K + FYM + LPSBF
T9	NP_100_K + FYM + CCB
T10	NP_100_K
T11	NP_100_K + LPSBF
T12	NP_100_K + CCB

Farmyard manure (FYM) @494 q ha^−1^ (20 t acre^−1^) was incorporated into the treatments with FYM before sowing. The sowing was done on 20 October 2022 and 23 October 2023, maintaining a row spacing of 60 cm and plant-to-plant spacing of 20 cm as per PAU's recommended practices. Recommended doses of nutrients including 185 kg of N (407.7 kg of urea), 61.8 kg of P_2_O_5_ (382 kg of single superphosphate) and 61.8 kg of K_2_O (98.8 kg of muriate of potash) per hectare were applied as a basal dose through urea, single super phosphate and muriate of potash, respectively. The half dose of urea and full doses of SSP and MOP were applied at sowing as per treatments. Another half dose of urea was applied at the time of earthing-up after sowing (Anonymous, 2025).

##### 2.6.2.1 Plant sample analysis

The data on plant growth parameters were recorded from 10 randomly selected plants in each replicate, and the mean values were estimated from three replicates. The haulm cutting was carried out manually during the second week of January and manually harvested in the last week of February during both the years. The data on emergence count was recorded at 15 days after sowing while all other parameters including shoot length, root length, fresh and dry weight, chlorophyll content, normalized difference vegetation index (NDVI), tuber number per plant, tuber yield, haulm (stem and leaves together) yield, nutrient content of haulm and tuber as well as soil parameters like pH, EC, organic carbon, available N, P, and K, microbial count, dehydrogenase and alkaline phosphatase were recorded at harvest. The tubers were graded into different size categories for yield assessment (Humagain et al., 2025).

Total phosphorus in plant samples and tubers were estimated using the vanado-molybdate yellow color method and total nitrogen was analyzed by the Kjeldahl method (Jackson, 1973). Potassium concentration in the filtrate was measured using a flame photometer (Model 1381).

##### 2.6.2.2 Soil sample analysis

Soil samples were collected at the start of the experiment and at harvest for analysis. Soil pH and electrical conductivity were determined in a 1:2.5 soil-to-water suspension and soil organic carbon was estimated by the rapid titration method (Jackson, 1973). Total nitrogen in soil was analyzed by the Kjeldahl method (Jackson, 1973). Available phosphorus was determined using the sodium bicarbonate extraction method (Olsen, 1954) and available potassium were analyzed by flame photometry. Rhizospheric soil samples were serially diluted up to 10^−7^ and plated on nutrient agar, Kenknight agar, potato dextrose agar, Jensen's agar, and modified Pikovskaya's agar for the enumeration of bacteria, actinomycetes, fungi, diazotrophs, and phosphate solubilizers, respectively.

Alkaline phosphatase activity was determined using the method described by Tabatabai and Bremner (1969). One gram of soil was placed in an Erlenmeyer flask, followed by the addition of 0.2 ml of toluene, 4 ml of modified universal buffer (pH 11 for alkaline phosphatase and pH 6.5 for acid phosphatase), and 1 ml of p-nitrophenyl phosphate (pNP) solution. The mixture was swirled briefly and incubated at 37 °C for 1 h. After incubation, 1 ml of 0.5 M CaCl_2_ and 4 ml of 0.5 M NaOH were added and mixed thoroughly. The soil suspension was then filtered using Whatman No. 1 filter paper, and the intensity of the yellow color in the filtrate was measured using a colorimeter at 405 nm. The values were expressed as μmol L^−1^ g^−1^ soil h^−1^ after calculating from the regression equation generated from the standard curve prepared from p-nitrophenol (pNP) standard solution.

Dehydrogenase activity was measured using the method described by Casida et al. (1964). One gram of soil was added to a 15 ml screw-cap test tube, along with 0.2 ml of 3% (w/v) Triphenyl tetrazolium chloride (TTC) and 0.5 ml of 1% (w/v) glucose. The contents were thoroughly mixed and incubated at 28 °C for 24 h. Following incubation, 10 ml of methanol was added, and the contents were mixed for 1 min. The tubes were then refrigerated for 3 h. The production of triphenyl formazan (TPF) was quantified by measuring absorbance at 485 nm after centrifugation. Dehydrogenase activity was calculated as μg of TPF per gram of soil per hour using the following formula:


$$\begin{array}{l} Dehydrogenase\;activity\;=\;C/Weight\;of\;soil\;sample\;\times \;24\\ \;=\;D\;ug\;TPF\;g^{-1}\;soil\;h^{-1} \end{array}$$

### 2.7 Statistical analysis

The experiments were laid out in a randomized block design with three replicates unless stated. The data were analyzed using two-way ANOVA with Tukey's-b *post hoc* test at 95% confidence intervals. Statistical analyses were performed using Statistix 10 software.

## 3 Results

### 3.1 Screening for phosphate solubilization

**Figure 2:**
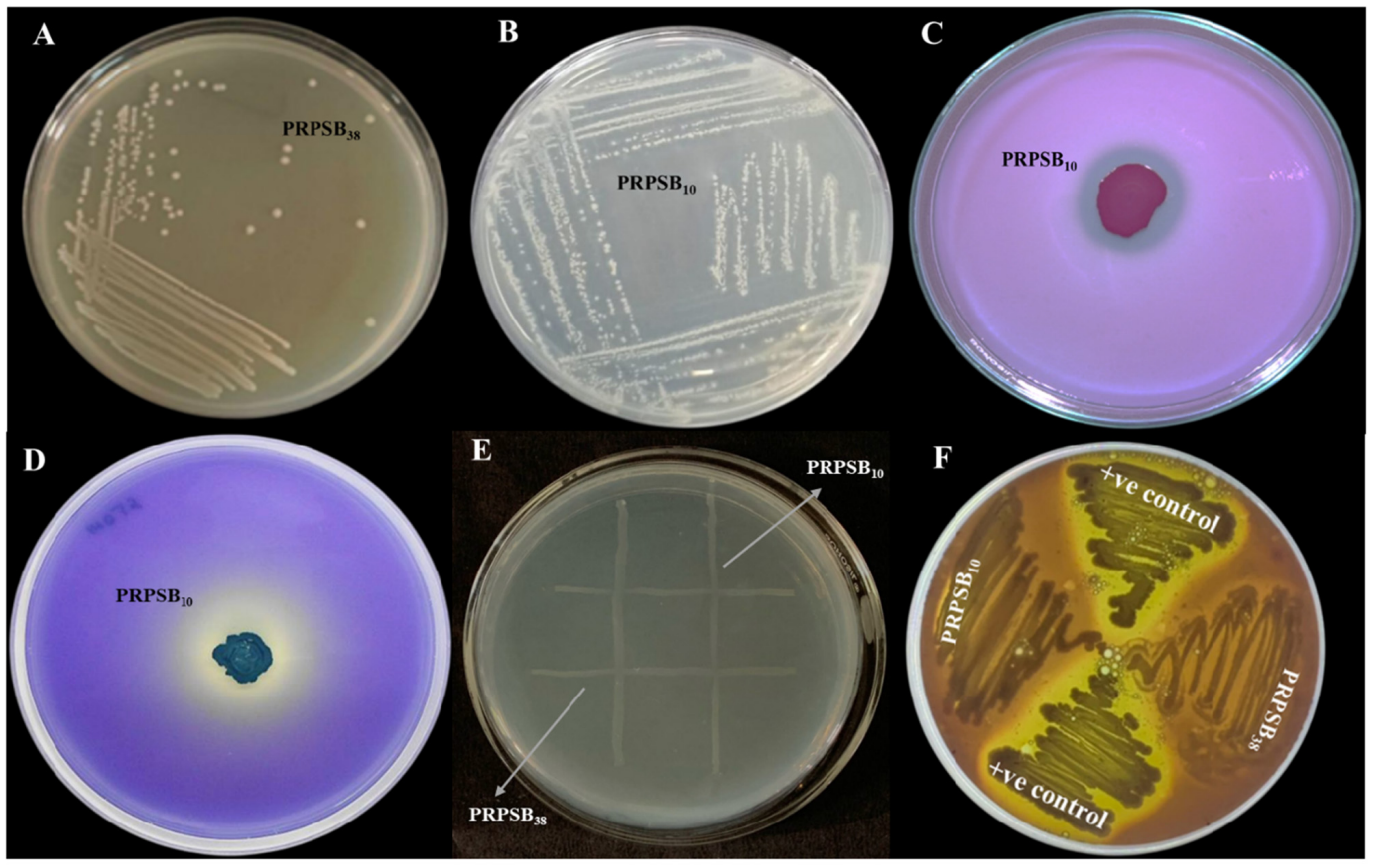
**(A, B)** Purified bacterial colonies on nutrient agar medium; **(C, D)** Qualitative screening of phosphate-solubilizing bacteria on modified Pikovskayas agar; **(E)** Compatibility testing of strains on nutrient agar by cross-streaked method; and **(F)** screening of phosphate-solubilizing bacteria for pathogenicity on blood agar.

Two phosphate-solubilizing bacterial strains *Bacillus licheniformis* PRPSB_10_ and *Pseudomonas putida* PRPSB_38_ isolated from potato rhizosphere and procured from the culture repository of the Department of Microbiology, Punjab Agricultural University, Ludhiana were tested for their phosphate-solubilizing ability (Figure 2). The strains PRPSB_10_ and PRPSB_38_ exhibited tricalcium phosphate solubilization index (PSI) of 6.1 and 5.8, respectively, on modified Pikovskaya (PVK) agar on fifth day of incubation (Figure 2).

Quantitative estimation in NBRIP broth revealed that both strains solubilized tricalcium phosphate (TCP), rock phosphate (RP), and Fe_3_(PO_4_)_2_, with TCP being the most efficiently solubilized substrate. The strain PRPSB_10_ solubilized 659.9, 202.0, and 113.7 μg ml^−1^ of TCP, RP, and Fe_3_(PO_4_)_2_, respectively, whereas PRPSB_38_ solubilized 457.4, 187.9, and 80.0 μg ml^−1^ of the same substrates, respectively, in NBRIP broth on fifth day of incubation. The results indicated that both the strains have retained their ability of solubilizing phosphate substrates.

### 3.2 Testing compatibility and pathogenicity of strains

To develop an effective bacterial bioformulation, it is essential to ensure the compatibility of the selected cultures. The compatibility between the PRPSB_10_ and PRPSB_38_ evaluated using the cross-streak method indicated that both the bacteria were able to grow with one another without exhibiting any signs of inhibition at the points of intersection along their streak lines, indicating that they were mutually compatible (Figure 2).

The strains did not show any zones of inhibition on blood agar, however the positive controls showed the zone of hemolysis on blood agar, indicating the non-pathogenic nature of the strains (Figure 2).

### 3.3 Development and testing shelf life of liquid formulation

The effect of three different cell protectants/additives, including trehalose, carboxymethyl cellulose (CMC), and polyethylene glycol (PEG) 6000, at three different concentrations was assessed on the viable cell count of the PRPSB_10_ and PRPSB_38_ over a storage period of 400 days.

#### 3.3.1 Formulation of Bacillus licheniformis (Bl) PRPSB_10_

**Figure 3:**
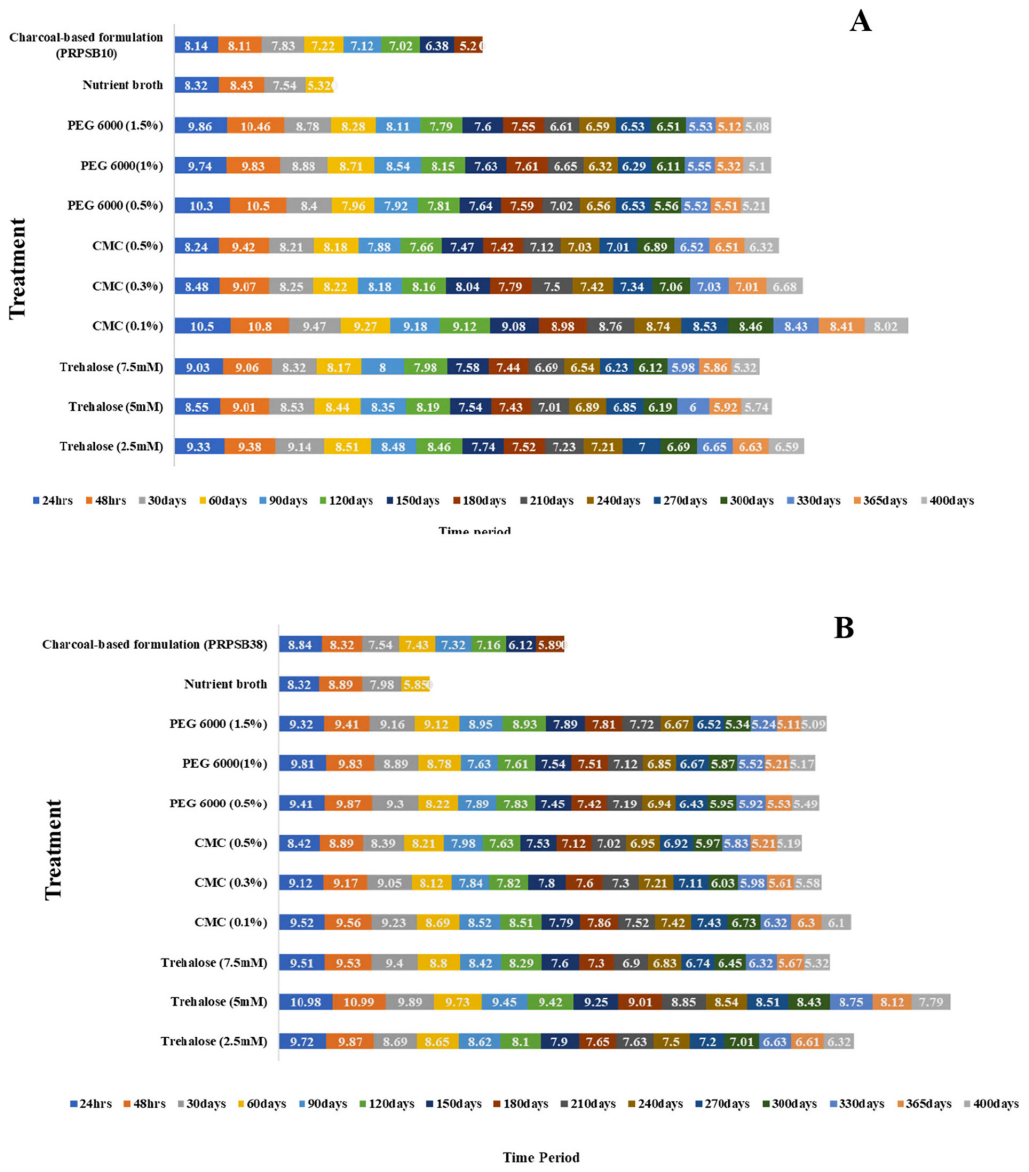
Viable count of phosphate-solubilizing *Bacillus licheniformis* PRPSB_10_ **(A)** and *Pseudomonas putida* PRPSB_38_ **(B)** in nutrient broth with different additives over a storage period of 400 days in comparison to charcoal-based formulation and unamended nutrient broth. Values are the mean of three replicates. Values in the bars represent Log_10_ CFU ml^−1^. PEG polyethylene glycol; CMC carboxymethyl cellulose.

Among the three concentrations of trehalose, 2.5 mM trehalose showed the highest viable count of 2.40 × 10^9^ CFU ml^−1^ after 48 h incubation, with the count gradually declining to 3.89 × 10^6^ CFU ml^−1^ after 365 days of storage (Figure 3). Likewise, at 5 mM and 7.5 mM concentrations of trehalose, the viable counts found after 365 days storage period were 5.50 × 10^5^ CFU ml^−1^ and 2.09 × 10^5^ CFU ml^−1^, respectively.

**Figure 4:**
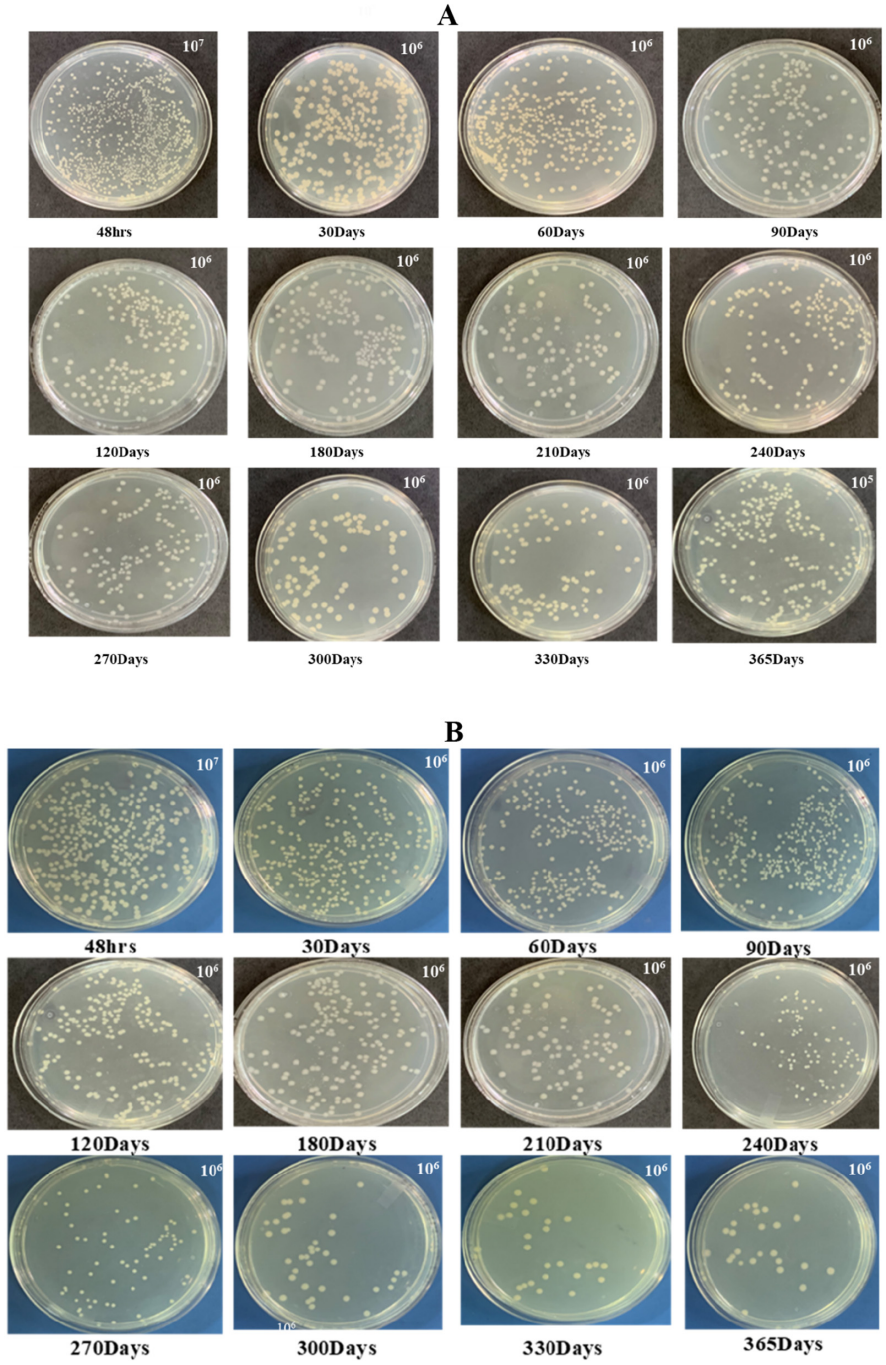
Viable count of phosphate-solubilizing *Bacillus licheniformis* PRPSB_10_ grown in nutrient broth amended with 0.1% carboxymethyl cellulose **(A)** and *Pseudomonas putida* PRPSB_38_ grown in nutrient broth with 5 mM trehalose **(B)** over a storage period of 365 days.

Similarly, among the three CMC concentrations, the highest viable count of *Bl* PRPSB_10_ was recorded at 0.1% concentration with the count increasing from 1.74 × 10^8^ at 24 h incubation to 6.31 × 10^10^ CFU ml^−1^ at 48 h incubation and then gradually reducing to 1.05 × 10^8^ CFU ml^−1^ after 1 year (Figures 3, 4). Similarly, at 0.3 and 0.5 concentrations of CMC, the count declined to 4.79 × 10^6^ CFU ml^−1^ and 2.09 × 10^6^ CFU ml^−1^ at 365 days, respectively, from the counts of 1.17 × 10^9^ CFU ml^−1^ and 2.63 × 10^9^ CFU ml^−1^, respectively, recorded at 48 h.

Likewise, among the PEG concentrations, the highest viable count observed in 0.5% PEG 6000 was 2.75 × 10^10^ CFU ml^−1^ after 48 h storage, which reduced to 1.62 × 10^5^ CFU ml^−1^ after 365 days of storage period (Figure 3). The viable counts observed at 1% PEG 6000 was 5.5 × 10^9^ CFU ml^−1^ after 48 h storage which declined to 1.6 × 10^5^ CFU ml^−1^ after 365 days, while the viable count in 1.5% PEG was found to be 7.24 × 10^9^ CFU ml^−1^ after 48 h reducing to 1.20 × 10^5^ CFU ml^−1^ after 365 days of storage.

The minimum count was recorded in the nutrient broth without any amendments. The highest viable count of *Bl*PRPSB_10_ in nutrient broth was found to be 2.69 × 10^8^ CFU ml^−1^ after 48 h of storage, which declined to 2.09 × 10^5^ CFU ml^−1^ at 60 days. Similarly, the viable count of *Bl*PRPSB_10_ in charcoal-based formulation was 1.38 × 10^8^ CFU ml^−1^ at 24 h, which reduced to 1.58 × 10^5^ CFU ml^−1^ by 180 days.

These results indicated that 0.1% CMC was the most effective additive for maintaining the long-term viability of *Bl*PRPSB_10_ in liquid formulation.

#### 3.3.2 Formulation of Pseudomonas putida (Pp) PRPSB_38_

Among the different concentrations of trehalose tested, the highest viable count of *Pp* PRPSB_38_ was observed at 5 mM concentration with the count increasing from 5.24 × 10^9^ at 24 h incubation to a maximum count of 7.94 × 10^10^ CFU ml^−1^ at 48 h incubation and then gradually decreasing to 6.17 × 10^7^ CFU ml^−1^ after 1 year storage (Figures 3, 4). At 2.5 mM trehalose concentration, the maximum viable count of 7.24 × 10^9^ CFU ml^−1^ was observed at 48 h storage which reduced to 2.09 × 10^6^ CFU ml^−1^ after 1 year. In the case of 7.5 mM trehalose, the count decreased to 2.09 × 10^5^ CFU ml^−1^ after 1 year of storage from a maximum count of 3.38 × 10^9^ CFU ml^−1^ after 48 h storage.

Similarly, within the different concentrations of CMC tested, 0.1% concentration supported the highest viable count of 3.63 × 10^9^ CFU ml^−1^ after 48 h, which gradually declined to 1.26 × 10^6^ CFU ml^−1^ after 1 year (Figure 3). At 0.3% and 0.5% CMC concentrations, the viable counts reduced to 3.80 × 10^5^ CFU ml^−1^ and 1.55 × 10^5^ CFU ml^−1^, respectively, after 365 days from the counts of 1.48 × 10^9^ CFU ml^−1^ and 7.76 × 10^8^ CFU ml^−1^, respectively, after 48 h storage.

Likewise, among the PEG concentrations, 0.5% resulted in the highest viable count of 7.41 × 10^9^ CFU ml^−1^ after 48 h, which reduced to 3.09 × 10^5^ CFU ml^−1^ after 1 year of storage. The viable counts at 1% and 1.5% PEG were 6.76 × 10^9^ CFU ml^−1^ and 2.57 × 10^9^ CFU ml^−1^ after 48 h which declined to 1.48 × 10^5^ CFU ml^−1^ and 1.23 × 10^5^ CFU ml^−1^, respectively, after 1 year of storage.

The minimum viable count of *Pp*PRPSB_38_ was observed in the nutrient broth without any additives with the highest viable count recorded to 7.76 × 10^8^ CFU ml^−1^ after 48 h of storage, which reduced to 7.08 × 10^5^ CFU ml^−1^ at 60 days. Likewise, the viable count of *Pp*PRPSB_38_ in charcoal-based biofertilizer was 6.92 × 10^8^ CFU ml^−1^ at 24 h, which declined to 3.89 × 10^5^ CFU ml^−1^ by 180 days.

These results indicated that 5 mM trehalose was the best additive for maintaining the long-term viability of *Pp*PRPSB_38_ in liquid formulation.

### 3.4 Evaluation of efficacy of PSB formulation in potato under field conditions

**Figure 5:**
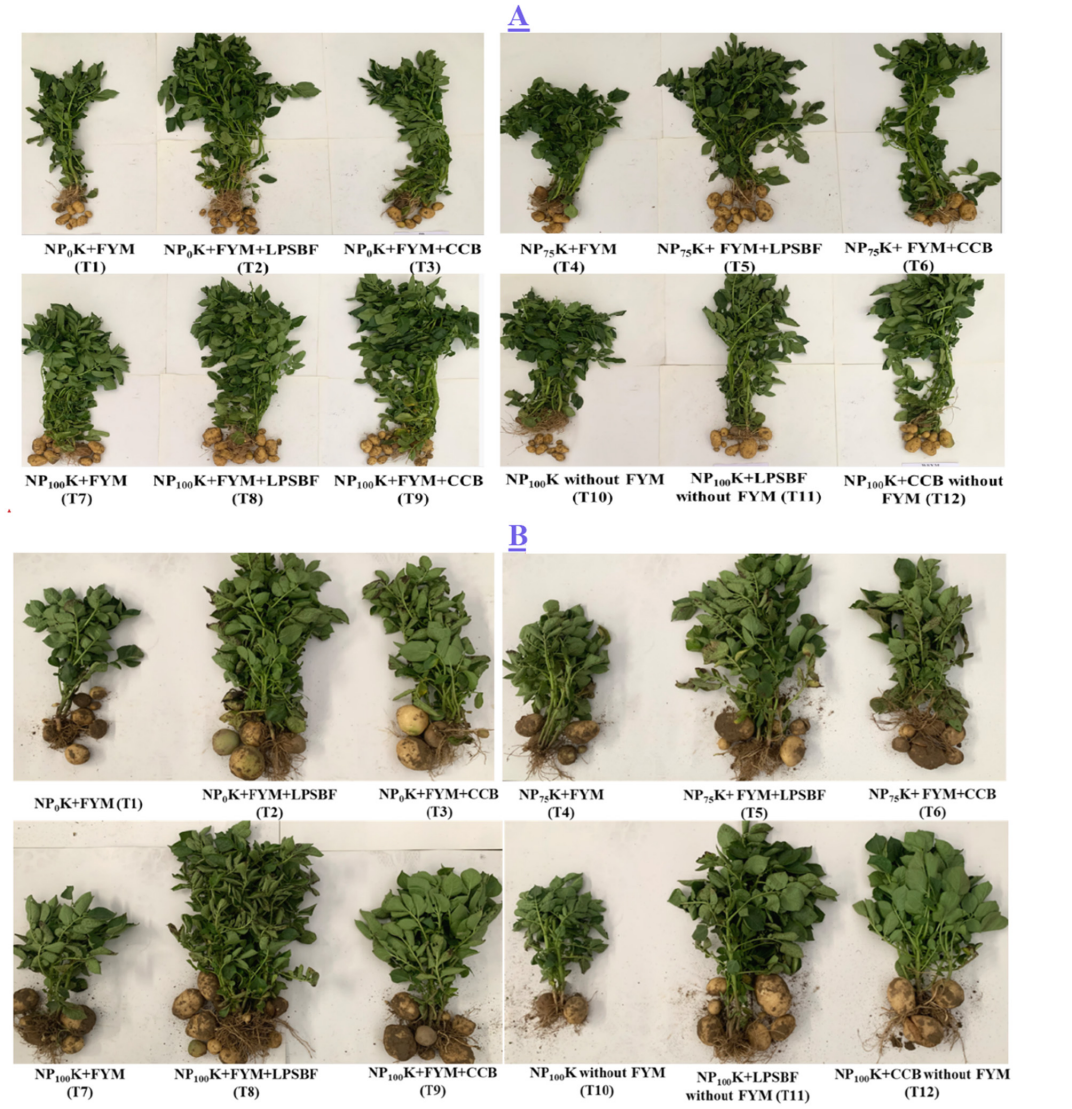
Effect of liquid formulation of *Bacillus licheniformis* PRPSB_10_ and *Pseudomonas putida* PRPSB_38_ on potato in the year 2022–23 **(A)** and 2023–24 **(B)**.

The efficacy of the liquid formulations of *Bl*PRPSB_10_ and *Pp*PRPSB_38_ (LPSBF) was assessed in potato under field conditions for two consecutive years in comparison to a charcoal-based consortium biofertilizer (CCB) recommended for potato by PAU (Anonymous, 2025) with and without farmyard manure (FYM) and 0%, 75%, and 100% dosage of P fertilizer (Figure 5). The climatic conditions of 2022–23 and 2023–24 at which the field trials were conducted are presented in Table 3.

**Table 3:** Climatic conditions during conduct of the field experiment.

**Year**	**Temperature (°C)**			**RH (%)**			**Rainfall**		**Sunshine (h)**
	**Max**	**Min**	**Mean**	**Morning**	**Evening**	**Mean**	**mm**	**Days**	
**2022–23**									
October	31.3	18.8	25.1	87	43	65	5.4	2	7.6
November	26.8	12.2	19.5	89	36	63	0	0	7.5
December	20.2	7.6	13.9	93	51	72	0.6	0	5.4
January	16.5	6.6	11.5	92	59	75	32.1	2	3.8
February	24.7	10.3	17.5	89	43	66	2.8	1	7.7
**2023–24**									
October	29.9	15.8	22.9	90	37	63	0	0	8.5
November	26.2	13.1	19.7	91	45	68	28.6	2	5.4
December	20.6	7.8	19.2	94	52	73	0	0	5.8
January	13.6	6.0	9.8	94	72	83	16.4	2	1.3
February	21.2	8.3	14.7	89	46	67	20.0	1	7.2

#### 3.4.1 Growth parameters

The liquid PSB formulation improved the growth parameters, nutrient content, yield parameters and soil available nutrients and enzymatic activities (Tables 4–10). The emergence count was improved, though the improvement was found to be non-significant compared to the uninoculated control treatments (Table 4). The emergence (%) ranged from 94.9 to 98.8% in the year 2022–23 and 95.2 to 99.1% during the year 2023–24 with the highest emergence recorded with the treatment T8 (NP_100_K + FYM + LPSBF), followed by T9 (NP_100_K + FYM + CCB), T7 (NP_100_K + FYM) and T5 (NP_75_K + FYM + LPSBF).

**Table 4:** Effect of liquid formulation of phosphate-solubilizing *Bacillus licheniformis* PRPSB_10_ and *Pseudomonas putida* PRPSB_38_ on growth of potato variety.

**Treatment**		**Emergence count (%)**			**Shoot length (cm)**			**Root length (cm)**			**Fresh weight (g plant** ^**−1**^ **)**			**Dry weight (g plant** ^**−1**^ **)**		
		**15 days after sowing**			**120 days after sowing**											
		**2022–23**	**2023–24**	**Mean**	**2022–23**	**2023–24**	**Mean**	**2022–23**	**2023–24**	**Mean**	**2022–23**	**2023–24**	**Mean**	**2022–23**	**2023–24**	**Mean**
T1	NP_0_K + FYM	94.9 ± 1.67	95.2 ± 1.94	95.0^a^	42.8 ± 0.2	48.0 ± 0.3	45.4^f^	14.5 ± 0.1	15.5 ± 0.1	15.0^h^	167.4 ± 0.5	168.6 ± 0.7	168.0^j^	8.2 ± 0.4	9.7 ± 0.3	8.9^i^
T2	NP_0_K + FYM + LPSBF	95.7 ± 0.94	95.9 ± 1.30	95.8^a^	47.2 ± 0.2	48.8 ± 0.6	48.0^f^	15.2 ± 0.1	16.0 ± 0.3	15.6^fgh^	180.3 ± 0.3	184.9 ± 0.8	182.7^h^	10.1 ± 0.2	12.5 ± 0.1	11.3^gh^
T3	NP_0_K + FYM + CCB	95.2 ± 1.01	95.3 ± 1.21	95.2^a^	45.7 ± 0.5	48.3 ± 0.2	47.0^f^	14.9 ± 0.1	15.9 ± 0.1	15.4^gh^	176.8 ± 0.4	180.2 ± 0.8	178.5^i^	9.7 ± 0.2	11.2 ± 0.1	10.5^h^
T4	NP_75_K + FYM	95.1 ± 0.89	97.8 ± 1.41	96.4^a^	52.9 ± 0.1	60.1 ± 0.1	56.5^d^	15.3 ± 0.3	17.2 ± 0.01	16.1^ef^	210.0 ± 0.8	213.0 ± 0.4	211.5^e^	13.4 ± 0.2	15.9 ± 0.3	14.6^e^
T5	NP_75_K + FYM + LPSBF	96.3 ± 1.10	97.9 ± 1.71	97.1^a^	56.1 ± 0.5	60.1 ± 1.4	58.1^c^	16.4 ± 0.1	21.0 ± 0.5	18.7^c^	220.1 ± 0.6	223.1 ± 0.2	221.6^d^	15.2 ± 0.3	16.3 ± 0.2	15.8^d^
T6	NP_75_K + FYM + CCB	95.5 ± 0.37	97.9 ± 1.50	96.7^a^	54.6 ± 0.8	60.4 ± 0.5	57.0^d^	17.5 ± 0.3	18.1 ± 0.1	17.8^d^	213.4 ± 0.4	217.6 ± 0.5	215.5^d^	15.4 ± 0.4	16.7 ± 0.3	16.1^cd^
T7	NP_100_K + FYM	97.1 ± 0.91	98.1 ± 1.21	97.6^a^	56.7 ± 0.1	63.3 ± 1.7	60.0^bc^	17.9 ± 0.3	23.0 ± 0.2	20.5^b^	237.3 ± 0.6	242.1 ± 0.8	239.7^c^	16.1 ± 0.1	18.0 ± 0.2	17.0^bc^
T8	NP_100_K + FYM + LPSBF	98.8 ± 0.55	99.1 ± 1.89	98.9^a^	65.2 ± 0.5	66.8 ± 1.1	66.0^a^	20.0 ± 0.4	24.0 ± 0.1	22.0^a^	265.0 ± 0.9	270.0 ± 0.7	267.5^a^	19.2 ± 0.2	19.9 ± 0.3	19.5^a^
T9	NP_100_K + FYM + CCB	97.4 ± 0.04	98.3 ± 0.04	97.8^a^	58.6 ± 1.2	64.4 ± 1.4	61.5^b^	19.3 ± 0.1	22.0 ± 0.5	20.6^b^	244.9 ± 0.3	250.1 ± 0.5	247.5^b^	16.8 ± 0.3	17.4 ± 0.2	17.1^b^
T10	NP_100_K without FYM	95.3 ± 0.08	96.6 ± 1.15	95.9^a^	49.7 ± 0.9	54.3 ± 0.5	52.0^e^	15.1 ± 0.3	17.0 ± 0.1	16.1^efg^	186.3 ± 0.6	189.1 ± 0.6	187.7^g^	11.1 ± 0.3	12.5 ± 0.7	11.8^fg^
T11	NP_100_K + LPSBF without FYM	96.1 ± 0.90	97.8 ± 1.95	96.9^a^	55.3 ± 0.1	59.7 ± 0.9	57.5^cd^	15.5 ± 0.3	18.0 ± 0.2	16.7^e^	207.9 ± 0.8	211.1 ± 0.3	209.5^e^	12.2 ± 0.1	15.3 ± 0.3	13.7^e^
T12	NP_100_K + CCB without FYM	95.9 ± 1.63	97.2 ± 0.74	96.5^a^	54.3 ± 0.3	57.7 ± 1.4	56.0^d^	15.7 ± 0.1	17.6 ± 0.2	16.6^e^	193.5 ± 0.5	199.5 ± 0.4	196.5^f^	12.2 ± 0.4	12.8 ± 0.3	12.5^f^
Mean		96.1^A^	97.3^A^		53.3^B^	57.7^A^		16.5^B^	18.8^A^		208.6^B^	212.4^A^		13.3^B^	14.8^A^	

Replications: 3, Design: RBD, Plot size: 5 m × 4 m; Date of sowing: 20.10.2022 and 23.10.2023. Values represent the mean of three replicates with 10 plants each. Values with different letters in a column differ significantly from one another with two-way ANOVA post hoc Tukey's-b test at *p* ≤ 0.05.

CCB, charcoal-based consortium biofertilizer; LPSBF, liquid phosphate-solubilizing bacterial formulation.

**Table 5:** Effect of liquid formulation of *Bacillus licheniformis* PRPSB_10_ and *Pseudomonas putida* PRPSB_38_ on growth and yield parameters potato grown in fields at harvest.

**Treatment**		**Chlorophyll content (SPAD value)**			**NDVI**			**Tuber number plant** ^**−1**^			**Tuber yield (q ha** ^**−1**^ **)**			**Haulm yield (q ha** ^**−1**^ **)**		
		**2022–23**	**2023–24**	**Mean**	**2022–23**	**2023–24**	**Mean**	**2022–23**	**2023–24**	**Mean**	**2022–23**	**2023–24**	**Mean**	**2022–23**	**2023–24**	**Mean**
T1	NP_0_K + FYM	42.8 ± 0.9	43.2 ± 0.3	43.0^e^	0.61	0.64	0.63^b^	5.0 ± 0.07	7.0 ± 0.13	6.0^i^	237.5 ± 0.41	245.5 ± 0.58	241^h^	178.0 ± 0.04	182.0 ± 0.28	180.0^f^
T2	NP_0_K + FYM + LPSBF	47.2 ± 0.4	47.5 ± 0.3	47.3^d^	0.66	0.74	0.70^b^	7.0 ± 0.01	9.0 ± 0.08	8.0^g^	261.0 ± 0.55	273.0 ± 0.66	267^d^	213.0 ± 0.30	213.5 ± 0.39	213.3^d^
T3	NP_0_K + FYM + CCB	44.0 ± 0.1	44.5 ± 0.4	44.2^e^	0.65	0.66	0.66^b^	7.0 ± 0.11	8.0 ± 0.17	7.5^h^	252.5 ± 0.28	257.5 ± 0.30	255^d^	194.0 ± 0.20	196.5 ± 0.25	195.3^e^
T4	NP_75_K + FYM	50.8 ± 0.5	51.1 ± 0.1	50.9^bc^	0.85	0.87	0.86^a^	9.0 ± 0.03	10.0 ± 0.23	9.5^de^	301.5 ± 0.96	313.0 ± 0.15	307^g^	216.0 ± 0.16	216.5 ± 0.37	216.3^cd^
T5	NP_75_K + FYM + LPSBF	51.6 ± 0.4	52.1 ± 0.6	51.3^abc^	0.93	0.95	0.94^a^	9.9 ± 0.22	10.7 ± 0.05	10.3^c^	308.5 ± 0.43	318.0 ± 0.42	313^cd^	217.5 ± 0.30	221.5 ± 0.09	219.5^c^
T6	NP_75_K + FYM + CCB	51.1 ± 0.5	51.3 ± 0.8	51.2^abc^	0.89	0.91	0.90^a^	9.0 ± 0.06	11.0 ± 0.13	10.0^cd^	305.8 ± 0.74	314.8 ± 0.82	310^cd^	214.5 ± 0.35	220.5 ± 0.40	217.5^cd^
T7	NP_100_K + FYM	51.3 ± 0.6	51.9 ± 0.9	51.6^abc^	0.94	0.95	0.95^a^	11.0 ± 0.13	110 ± 0.24	11.0^b^	346.5 ± 0.74	355.5 ± 0.82	351^b^	221.5 ± 0.07	223.5 ± 0.25	222.5^b^
T8	NP_100_K + FYM + LPSBF	53.3 ± 0.2	53.9 ± 0.8	53.6^a^	0.93	0.98	0.96^a^	12.0 ± 0.17	13.0 ± 0.28	12.5^a^	396.5 ± 0.81	420.0 ± 1.25	408^a^	228.5 ± 0.41	232.5 ± 0.08	230.5^a^
T9	NP_100_K + FYM + CCB	52.1 ± 0.1	53.1 ± 0.3	52.6^ab^	0.9	0.96	0.93^a^	11.0 ± 0.20	12.0 ± 0.19	11.5^b^	376.0 ± 0.12	384.0 ± 1.11	380^a^	226.0 ± 0.24	227.0 ± 0.16	226.5^ab^
T10	NP_100_K without FYM	49.3 ± 0.6	49.4 ± 0.6	49.3^cd^	0.75	0.8	0.78^a^	7.0 ± 0.04	9.0 ± 0.02	8.0^gh^	297.5 ± 0.12	310.0 ± 0.67	303^de^	216.0 ± 0.18	210.5 ± 0.44	213.3^d^
T11	NP_100_K + LPSBF without FYM	49.6 ± 0.4	50.02 ± 0.3	49.8^cd^	0.83	0.81	0.82^a^	8.0 ± 0.02	10.0 ± 0.18	9.0^ef^	300.8 ± 0.11	312.3 ± 0.17	306^d^	218.0 ± 0.10	213.0 ± 0.03	215.5^cd^
T12	NP_100_K + CCB without FYM	49.5 ± 0.3	49.8 ± 0.4	49.6^cd^	0.79	0.8	0.80^a^	8.0 ± 0.19	9.0 ± 0.11	8.5^fg^	298.8 ± 0.71	310.3 ± 0.82	304^de^	214.5 ± 0.32	214.0 ± 0.30	214.3^d^
Mean		49.2^A^	49.7^A^		0.81^A^	0.84^A^		8.74^B^	9.98^A^		307^B^	318^A^		213.1^B^	214.2^A^	

Replications: 3, Design: RBD, Plot size: 5 m × 4 m; Date of sowing: 20.10.2022 and 23.10.2023. Values represent the mean of three replicates with 10 plants each. Values with different letters in a column differ significantly from one another with two-way ANOVA post hoc Tukey's-b test at *p* ≤ 0.05.

NDVI, Normalized difference vegetation index; CCB, charcoal-based consortium biofertilizer; LPSBF, liquid phosphate-solubilizing bacterial formulation. One q = 100 kg.

**Table 6:** Effect of liquid formulation of *Bacillus licheniformis* PRPSB_10_ and *Pseudomonas putida* PRPSB_38_ on grading of potato grown in fields at harvest.

**Treatment**		**Yield (q ha** ^**−1**^ **)**								
		**Tuber size 25–35 mm**			**Tuber size 45–55 mm**			**Tuber size** >**55 mm**		
		**2022–23**	**2023–24**	**Mean**	**2022–23**	**2023–24**	**Mean**	**2022–23**	**2023–24**	**Mean**
T1	NP_0_K + FYM	50.8 ± 0.01	51.5 ± 0.04	51.1^e^	57.7 ± 0.01	59.7 ± 0.08	58.7^d^	129.1 ± 0.25	134.7 ± 0.27	131.9^f^
T2	NP_0_K + FYM + LPSBF	57.8 ± 0.01	60.1 ± 0.02	58.9^d^	65.1 ± 0.01	60.0 ± 0.08	62.5^d^	138.1 ± 0.44	149.9 ± 0.47	144.1^e^
T3	NP_0_K + FYM + CCB	53.1 ± 0.04	54.1 ± 0.04	53.6^e^	62.3 ± 0.03	59.9 ± 0.05	61.1^d^	137.3 ± 0.06	143.7 ± 0.13	140.5^ef^
T4	NP_75_K + FYM	48.8 ± 0.06	52.2 ± 0.08	50.5^e^	81.5 ± 0.17	83.7 ± 0.2	82.6^bc^	168.5 ± 0.07	170.5 ± 0.48	169.5^d^
T5	NP_75_K + FYM + LPSBF	53.8 ± 0.15	56.2 ± 0.09	55.0^de^	86.2 ± 0.24	91.2 ± 0.18	88.7^ab^	171.2 ± 0.58	177.0 ± 0.24	174.1^cd^
T6	NP_75_K + FYM + CCB	51.0 ± 0.03	54.7 ± 0.16	52.8^e^	82.5 ± 0.12	80.2 ± 0.19	81.3^bc^	172.2 ± 0.62	179.7 ± 0.58	176.0^cd^
T7	NP_100_K + FYM	77.7 ± 0.07	80.9 ± 0.10	79.3^b^	87.7 ± 0.07	88.1 ± 0.01	87.9^ab^	182.7 ± 0.31	200.7 ± 0.46	191.7^bc^
T8	NP_100_K + FYM + LPSBF	80.7 ± 0.15	91.3 ± 0.21	86.1^a^	84.2 ± 0.1	98.5 ± 0.04	91.4^a^	220.7 ± 0.54	209.7 ± 0.28	215.2^a^
T9	NP_100_K + FYM + CCB	79.2 ± 0.13	88.5 ± 0.19	83.9^a^	83.9 ± 0.07	97.8 ± 0.16	90.9^a^	213.1 ± 0.55	197.9 ± 0.57	205.5^ab^
T10	NP_100_K without FYM	40.5 ± 0.05	42.5 ± 0.05	41.5^f^	72.0 ± 0.05	77.5 ± 0.05	74.7^c^	177.5 ± 0.46	188.0 ± 0.64	182.7^c^
T11	NP_100_K + LPSBF without FYM	45.2 ± 0.01	50.5 ± 0.04	47.8^ef^	78.0 ± 0.18	73.7 ± 0.14	75.8^c^	185.0 ± 0.13	190.0 ± 0.14	187.5^c^
T12	NP_100_K + CCB without FYM	43.2 ± 0.11	46.2 ± 0.05	44.7^f^	72.5 ± 0.03	75.0 ± 0.01	73.7^c^	183.0 ± 0.17	189.0 ± 0.7	186.0^c^
Mean		56.8^A^	60.8^A^		76.2^B^	78.8^A^		173.2^B^	177.6^A^	

Replications: 3, Design: RBD, Plot size: 5 m × 4 m; Date of sowing: 20.10.2022 and 23.10.2023. Values represent the mean of three replicates. Values with different letters in a column differ significantly from one another with two-way ANOVA post hoc Tukey's-b test at *p* ≤ 0.05.

CCB, charcoal-based consortium biofertilizer; LPSBF, liquid phosphate-solubilizing bacterial formulation. One q = 100 kg.

**Table 7:** Effect of liquid formulation of *Bacillus licheniformis* PRPSB_10_ and *Pseudomonas putida* PRPSB_38_ on nutrient content of haulm and tuber of potato grown in field at harvest.

**Treatment**		**N**			**P**			**K**		
		**2022–23**	**2023–24**	**Mean**	**2022–23**	**2023–24**	**Mean**	**2022–23**	**2023–24**	**Mean**
**Nutrient content of tuber (%)**										
T1	NP_0_K + FYM	1.40 ± 0.022	1.42 ± 0.036	1.41^e^	0.25 ± 0.006	0.26 ± 0.003	0.25^i^	1.45 ± 0.029	1.47 ± 0.013	1.46^d^
T2	NP_0_K + FYM + LPSBF	1.43 ± 0.026	1.49 ± 0.031	1.46^de^	0.29 ± 0.004	0.31 ± 0.005	0.30^gh^	1.49 ± 0.012	1.50 ± 0.025	1.50^cd^
T3	NP_0_K + FYM + CCB	1.45 ± 0.008	1.46 ± 0.011	1.45^de^	0.29 ± 0.004	0.28 ± 0.007	0.29^h^	1.48 ± 0.002	1.49 ± 0.018	1.49^cd^
T4	NP_75_K + FYM	1.45 ± 0.002	1.53 ± 0.005	1.49^d^	0.38 ± 0.003	0.39 ± 0.002	0.38^e^	1.51 ± 0.031	1.52 ± 0.014	1.52^cd^
T5	NP_75_K + FYM + LPSBF	1.52 ± 0.002	1.53 ± 0.024	1.53^cd^	0.42 ± 0.001	0.45 ± 0.001	0.43^c^	1.54 ± 0.035	1.55 ± 0.035	1.55^bc^
T6	NP_75_K + FYM + CCB	1.51 ± 0.039	1.53 ± 0.029	1.52^cd^	0.40 ± 0.003	0.42 ± 0.003	0.41^d^	1.54 ± 0.010	1.55 ± 0.005	1.55^bc^
T7	NP_100_K + FYM	1.56 ± 0.002	1.59 ± 0.007	1.58^bc^	0.44 ± 0.005	0.45 ± 0.011	0.44^c^	1.55 ± 0.012	1.56 ± 0.027	1.56^bc^
T8	NP_100_K + FYM + LPSBF	1.67 ± 0.041	1.68 ± 0.003	1.68^a^	0.51 ± 0.001	0.53 ± 0.010	0.52^a^	1.65 ± 0.035	1.67 ± 0.002	1.66^a^
T9	NP_100_K + FYM + CCB	1.59 ± 0.002	1.61 ± 0.026	1.60^b^	0.49 ± 0.005	0.50 ± 0.012	0.50^b^	1.60 ± 0.036	1.61 ± 0.035	1.61^ab^
T10	NP_100_K without FYM	1.48 ± 0.025	1.47 ± 0.007	1.48^de^	0.31 ± 0.005	0.33 ± 0.008	0.32^fg^	1.50 ± 0.012	1.51 ± 0.030	1.51^cd^
T11	NP_100_K + LPSBF without FYM	1.49 ± 0.022	1.50 ± 0.006	1.50^d^	0.32 ± 0.003	0.34 ± 0.007	0.33^f^	1.54 ± 0.015	1.52 ± 0.032	1.53^bcd^
T12	NP_100_K + CCB without FYM	1.48 ± 0.012	1.49 ± 0.006	1.49^d^	0.32 ± 0.007	0.32 ± 0.006	0.32^fg^	1.51 ± 0.004	1.52 ± 0.019	1.52^cd^
Mean		1.50^A^	1.52^A^		0.37^B^	0.38^A^		1.53^A^	1.54^A^	
**Nutrient content of haulm (%)**										
T1	NP_0_K + FYM	1.04 ± 0.01	1.05 ± 0.01	1.05^f^	0.15 ± 0.003	0.18 ± 0.006	0.17^h^	1.2 ± 0.017	1.24 ± 0.02	1.22^h^
T2	NP_0_K + FYM + LPSBF	1.07 ± 0.02	1.08 ± 0.01	1.08^ef^	0.21 ± 0.001	0.22 ± 0.005	0.22^f^	1.36 ± 0.001	1.37 ± 0.022	1.37^fg^
T3	NP_0_K + FYM + CCB	1.05 ± 0.02	1.06 ± 0.03	1.06^f^	0.21 ± 0.002	0.19 ± 0.004	0.20^g^	1.32 ± 0.024	1.28 ± 0.021	1.30^g^
T4	NP_75_K + FYM	1.15 ± 0.03	1.17 ± 0.03	1.16^cd^	0.29 ± 0.007	0.30 ± 0.006	0.30^c^	1.45 ± 0.016	1.49 ± 0.032	1.47^cd^
T5	NP_75_K + FYM + LPSBF	1.19 ± 0.03	1.21 ± 0.01	1.20^bc^	0.30 ± 0.005	0.31 ± 0.004	0.31^c^	1.49 ± 0.003	1.53 ± 0.013	1.51^bcd^
T6	NP_75_K + FYM + CCB	1.15 ± 0.03	1.16 ± 0.01	1.16^cd^	0.30 ± 0.003	0.31 ± 0.001	0.31^c^	1.49 ± 0.013	1.51 ± 0.01	1.50^bcd^
T7	NP_100_K + FYM	1.21 ± 0.02	1.23 ± 0.01	1.22^b^	0.31 ± 0.002	0.32 ± 0.005	0.32^c^	1.51 ± 0.014	1.53 ± 0.007	1.52^bc^
T8	NP_100_K + FYM + LPSBF	1.28 ± 0.01	1.29 ± 0.01	1.29^a^	0.38 ± 0.007	0.39 ± 0.008	0.39^a^	1.68 ± 0.039	1.7 ± 0.019	1.69^a^
T9	NP_100_K + FYM + CCB	1.24 ± 0.03	1.26 ± 0.01	1.25^ab^	0.32 ± 0.001	0.34 ± 0.002	0.33^b^	1.55 ± 0.036	1.60 ± 0.016	1.58^b^
T10	NP_100_K without FYM	1.08 ± 0.02	1.09 ± 0.01	1.09^ef^	0.23 ± 0.003	0.25 ± 0.003	0.24^f^	1.37 ± 0.017	1.41 ± 0.034	1.39^ef^
T11	NP_100_K + LPSBF without FYM	1.12 ± 0.01	1.14 ± 0.01	1.13^de^	0.27 ± 0.003	0.29 ± 0.002	0.28^d^	1.43 ± 0.013	1.47 ± 0.002	1.45^cde^
T12	NP_100_K + CCB without FYM	1.09 ± 0.01	1.10 ± 0.01	1.10^ef^	0.26 ± 0.006	0.27 ± 0.004	0.27^e^	1.43 ± 0.028	1.45 ± 0.037	1.44^def^
		1.14^A^	1.15^A^		0.27^A^	0.27^A^		1.46^A^	1.45^A^	

Replications: 3, Design: RBD, Plot size: 5 m × 4 m; Date of sowing: 20.10.2022 and 23.10.2023. Values represent the mean of three replicates. Values with different letters in a column differ significantly from one another with two-way ANOVA post hoc Tukey's-b test at *p* ≤ 0.0.5.

CCB, charcoal-based consortium biofertilizer; LPSBF, liquid phosphate-solubilizing bacterial formulation.

**Table 8:** Effect of liquid formulation of *Bacillus licheniformis* PRPSB_10_ and *Pseudomonas putida* PRPSB_38_ on soil parameters of potato at harvest in field.

**Treatment**		**pH**			**EC (dS m** ^**−1**^ **)**			**Organic carbon (%)**			**Available N (kg ha** ^**−1**^ **)**			**Available P (kg ha** ^**−1**^ **)**			**Available K (kg ha** ^**−1**^ **)**		
		**2022–23**	**2023–24**	**Mean**	**2022–23**	**2023–24**	**Mean**	**2022–23**	**2023–24**	**Mean**	**2022–23**	**2023–24**	**Mean**	**2022–23**	**2023–24**	**Mean**	**2022–23**	**2023–24**	**Mean**
T1	NP_0_K + FYM	7.45 ± 0.05	7.94 ± 0.08	7.69^a^	0.247 ± 0.002	0.267 ± 0.002	0.257^a^	0.41 ± 0.001	0.42 ± 0.002	0.42^c^	166.9 ± 0.4	167.1 ± 1.3	167.0^d^	16.5 ± 0.22	17.9 ± 0.01	17.2^e^	169.7 ± 1.67	170.1 ± 0.60	169.90^c^
T2	NP_0_K + FYM + LPSBF	7.40 ± 0.08	7.92 ± 0.07	7.66^a^	0.249 ± 0.005	0.271 ± 0.001	0.260^a^	0.42 ± 0.003	0.43 ± 0.002	0.43^b^	168.9 ± 1.5	169.9 ± 1.1	169.4^d^	17.8 ± 0.09	18.8 ± 0.19	18.3^de^	170.9 ± 0.58	171.6 ± 0.44	171.25^c^
T3	NP_0_K + FYM + CCB	7.43 ± 0.12	7.91 ± 0.08	7.67^a^	0.248 ± 0.004	0.268 ± 0.005	0.258^a^	0.42 ± 0.002	0.43 ± 0.005	0.43^b^	168.2 ± 1.3	169.8 ± 0.6	169.0^d^	17.5 ± 0.13	18.4 ± 0.19	18.0^de^	170.4 ± 1.34	171.0 ± 1.08	170.70^c^
T4	NP_75_K + FYM	7.43 ± 0.15	7.93 ± 0.09	7.68^a^	0.248 ± 0.002	0.268 ± 0.005	0.258^a^	0.43 ± 0.002	0.44 ± 0.003	0.44^a^	180.5 ± 1.1	181.2 ± 1.8	180.8^c^	19.7 ± 0.15	22.9 ± 0.38	21.3^c^	183.8 ± 2.23	184.9 ± 1.34	184.35^ab^
T5	NP_75_K + FYM + LPSBF	7.41 ± 0.06	7.84 ± 0.02	7.63^a^	0.251 ± 0.004	0.272 ± 0.001	0.262^a^	0.44 ± 0.005	0.45 ± 0.006	0.45^a^	182.4 ± 0.3	182.9 ± 1.4	182.6^abc^	21.8 ± 0.22	23.7 ± 0.22	22.8^b^	185.2 ± 1.21	185.3 ± 1.64	185.25^ab^
T6	NP_75_K + FYM + CCB	7.42 ± 0.01	7.83 ± 0.13	7.63^a^	0.248 ± 0.01	0.269 ± 0.002	0.258^a^	0.43 ± 0.004	0.44 ± 0.005	0.44^a^	181.7 ± 1.0	182.9 ± 1.6	182.3^abc^	21.1 ± 0.41	22.4 ± 0.23	21.8^c^	183.8 ± 0.94	185.2 ± 1.24	184.52^ab^
T7	NP_100_K + FYM	7.42 ± 0.11	7.91 ± 0.16	7.67^a^	0.253 ± 0.004	0.269 ± 0.005	0.261^a^	0.44 ± 0.007	0.45 ± 0.002	0.45^a^	183.5 ± 1.2	184.4 ± 1.3	183.9^ab^	21.9 ± 0.33	23.9 ± 0.31	22.9^b^	185.3 ± 0.58	186.8 ± 1.58	186.05^ab^
T8	NP_100_K + FYM + LPSBF	7.39 ± 0.12	7.89 ± 0.14	7.64^a^	0.252 ± 0.005	0.272 ± 0.004	0.262^a^	0.45 ± 0.005	0.46 ± 0.01	0.46^a^	185.5 ± 1.5	189.2 ± 1.3	187.3^a^	23.8 ± 0.28	26.7 ± 0.19	25.2^a^	187.6 ± 0.96	189.9 ± 1.86	188.75^a^
T9	NP_100_K + FYM + CCB	7.41 ± 0.06	7.90 ± 0.02	7.65^a^	0.251 ± 0.004	0.270 ± 0.005	0.260^a^	0.44 ± 0.008	0.45 ± 0.009	0.45^a^	183.3 ± 1.3	185.4 ± 0.4	184.3^ab^	22.1 ± 0.41	25.9 ± 0.46	24.0^b^	185.8 ± 1.40	186.9 ± 1.74	186.35^ab^
T10	NP_100_K without FYM	7.42 ± 0.10	7.89 ± 0.08	7.65^a^	0.249 ± 0.003	0.268 ± 0.004	0.258^a^	0.34 ± 0.001	0.36 ± 0.009	0.35^e^	168.8 ± 1.6	170.5 ± 1.9	169.6^d^	17.9 ± 0.35	19.3 ± 0.22	18.6^d^	172.9 ± 1.11	173.6 ± 1.08	173.25^c^
T11	NP_100_K + LPSBF without FYM	7.41 ± 0.13	7.88 ± 0.10	7.64^a^	0.251 ± 0.003	0.271 ± 0.004	0.261^a^	0.39 ± 0.004	0.40 ± 0.002	0.39^d^	178.5 ± 1.4	179.1 ± 1.4	178.8^bc^	20.7 ± 0.23	21.5 ± 0.42	21.1^c^	183.4 ± 1.44	184.0 ± 1.56	183.70^ab^
T12	NP_100_K + CCB without FYM	7.41 ± 0.14	7.85 ± 0.11	7.63^a^	0.250 ± 0.001	0.268 ± 0.001	0.259^a^	0.36 ± 0.001	0.38 ± 0.004	0.37^e^	177.2 ± 1.8	178.2 ± 0.9	177.7^c^	18.3 ± 0.08	19.4 ± 0.38	18.8^d^	180.7 ± 1.38	181.2 ± 0.80	180.95^b^
Mean		7.41^B^	7.89^A^		0.25^A^	0.27^A^		0.41^B^	0.43^A^		177.1^A^	178.4^A^		19.8^B^	21.9^A^		179.9^A^	180.8^A^	

Replications: 3, Design: RBD, Plot size: 5 m × 4 m; Date of sowing: 20.10.2022 and 23.10.2023. Values represent the mean of three replicates with 10 plants each. Values with different letters in a column differ significantly from one another with two-way ANOVA post hoc Tukey's-b test at *p* ≤ 0.05.

CCB, charcoal-based consortium biofertilizer; LPSBF, liquid phosphate-solubilizing bacterial formulation.

Initial soil properties of the field trial during 2022–23 and 2023–24 were as follows: pH 7.60 and 7.95; EC 0.22 and 0.24 dS m^−1^; OC 0.39 and 0.40%; available N 165.4 and 165.9 kg ha^−1^; available P 15.7 and 16.3 kg ha^−1^; and available K 168.5 and 169.7 kg ha^−1^, respectively.

**Table 9:** Effect of liquid formulation of *Bacillus licheniformis* PRPSB_10_ and *Pseudomonas putida* PRPSB_38_ on microbial count in the rhizosphere of potato grown in fields at harvest.

**Treatment**		**Microbial count (Log**_**10**_ **CFU g**^**−1**^**)**														
		**Bacteria**			**Fungi**			**P-solubilizers**			**Actinomycetes**			**Diazotrophs**		
		**2022–23**	**2023–24**	**Mean**	**2022–23**	**2023–24**	**Mean**	**2022–23**	**2023–24**	**Mean**	**2022–23**	**2023–24**	**Mean**	**2022–23**	**2023–24**	**Mean**
T1	NP_0_K + FYM	8.31 ± 0.15	8.37 ± 0.14	8.34^a^	3.90 ± 0.06	3.95 ± 0.07	3.93^a^	7.20 ± 0.04	7.36 ± 0.12	7.28^a^	7.24 ± 0.03	7.38 ± 0.09	7.31^a^	6.01 ± 0.11	6.11 ± 0.02	6.06^a^
T2	NP_0_K + FYM + LPSBF	8.43 ± 0.15	8.54 ± 0.01	8.49^a^	4.10 ± 0.01	4.18 ± 0.04	4.14^a^	7.30 ± 0.01	7.44 ± 0.02	7.37^a^	7.52 ± 0.10	7.42 ± 0.02	7.47^a^	6.70 ± 0.13	6.80 ± 0.01	6.75^a^
T3	NP_0_K + FYM + CCB	8.71 ± 0.04	8.68 ± 0.09	8.70^a^	4.20 ± 0.08	4.26 ± 0.03	4.23^a^	7.20 ± 0.05	7.30 ± 0.08	7.25^a^	7.45 ± 0.10	7.73 ± 0.03	7.59^a^	6.40 ± 0.07	6.50 ± 0.07	6.45^a^
T4	NP_75_K + FYM	8.36 ± 0.17	8.54 ± 0.02	8.45^a^	4.00 ± 0.01	4.06 ± 0.01	4.03^a^	7.41 ± 0.03	7.50 ± 0.14	7.46^a^	7.38 ± 0.07	7.37 ± 0.04	7.38^a^	6.23 ± 0.13	6.33 ± 0.01	6.28^a^
T5	NP_75_K + FYM + LPSBF	8.49 ± 0.13	8.62 ± 0.12	8.56^a^	4.20 ± 0.04	4.23 ± 0.08	4.22^a^	7.76 ± 0.14	7.80 ± 0.06	7.78^a^	7.65 ± 0.15	7.49 ± 0.11	7.57^a^	6.65 ± 0.04	6.75 ± 0.05	6.70^a^
T6	NP_75_K + FYM + CCB	8.81 ± 0.11	8.75 ± 0.05	8.78^a^	4.10 ± 0.01	4.11 ± 0.08	4.11^a^	7.71 ± 0.07	7.70 ± 0.10	7.71^a^	7.74 ± 0.02	7.66 ± 0.02	7.70^a^	6.90 ± 0.10	7.00 ± 0.01	6.95^a^
T7	NP_100_K + FYM	8.42 ± 0.14	8.57 ± 0.03	8.50^a^	4.10 ± 0.05	4.14 ± 0.05	4.12^a^	7.40 ± 0.03	7.63 ± 0.14	7.52^a^	7.53 ± 0.10	7.63 ± 0.04	7.58^a^	6.32 ± 0.05	6.42 ± 0.07	6.37^a^
T8	NP_100_K + FYM + LPSBF	8.70 ± 0.14	8.86 ± 0.05	8.78^a^	4.30 ± 0.03	4.39 ± 0.01	4.35^a^	7.93 ± 0.01	7.95 ± 0.02	7.94^a^	7.89 ± 0.05	7.86 ± 0.12	7.88^a^	6.62 ± 0.11	6.72 ± 0.07	6.67^a^
T9	NP_100_K + FYM + CCB	8.85 ± 0.15	8.81 ± 0.12	8.83^a^	4.20 ± 0.04	4.25 ± 0.05	4.23^a^	7.78 ± 0.01	7.86 ± 0.13	7.82^a^	7.85 ± 0.05	7.84 ± 0.13	7.85^a^	6.92 ± 0.14	7.02 ± 0.13	6.97^a^
T10	NP_100_K without FYM	8.40 ± 0.05	8.51 ± 0.03	8.46^a^	3.90 ± 0.07	3.91 ± 0.07	3.91^a^	7.57 ± 0.05	7.27 ± 0.15	7.42^a^	7.60 ± 0.14	7.39 ± 0.14	7.50^a^	6.31 ± 0.06	6.41 ± 0.03	6.36^a^
T11	NP_100_K + LPSBF without FYM	8.71 ± 0.10	8.68 ± 0.08	8.70^a^	4.20 ± 0.04	4.27 ± 0.02	4.24^a^	7.83 ± 0.03	7.71 ± 0.12	7.77^a^	7.67 ± 0.03	7.81 ± 0.11	7.74^a^	6.34 ± 0.10	6.44 ± 0.09	6.39^a^
T12	NP_100_K + CCB without FYM	8.77 ± 0.02	8.74 ± 0.08	8.76^a^	4.10 ± 0.06	4.14 ± 0.03	4.12^a^	7.62 ± 0.13	7.84 ± 0.09	7.73^a^	7.53 ± 0.05	7.61 ± 0.13	7.57^a^	6.95 ± 0.11	7.05 ± 0.01	7.00^a^
Mean		8.50^A^	8.64^A^		4.11^A^	4.16^A^		7.54^A^	7.57^A^		7.59^A^	7.58^A^		6.53^A^	6.61^A^	

Replications: 3, Design: RBD, Plot size: 5 m × 4 m; Date of sowing: 20.10.2022 and 23.10.2023. Values represent the mean of three replicates with 10 plants each. Values with different letters in a column differ significantly from one another with one-way ANOVA post hoc Tukey's-b test at *p* ≤ 0.05.

CCB, charcoal-based consortium biofertilizer; LPSBF, liquid phosphate-solubilizing bacterial formulation; DAS, days after sowing.

**Table 10:** Effect of liquid formulation of *Bacillus licheniformis* PRPSB_10_ and *Pseudomonas putida* PRPSB_38_ on soil enzymatic activities and economics of potato cultivation.

**Treatment**		**Alkaline phosphatase activity (**μ**g pNP g**^**−1**^ **h**^**−1**^**)**			**Dehydrogenase activity (**μ**g TPF g**^**−1**^ **h**^**−1**^**)**			**Gross income (**₹ **ha**^**−1**^**)**			**Net income (**₹ **ha**^**−1**^**)**			**B/C ratio**		
		**2022–23**	**2023–24**	**Mean**	**2022–23**	**2023–24**	**Mean**	**2022–23**	**2023–24**	**Mean**	**2022–23**	**2023–24**	**Mean**	**2022–23**	**2023–24**	**Mean**
T1	NP_0_K + FYM	22.8 ± 0.3	23.7 ± 0.6	23.3^f^	44.5 ± 0.5	45.3 ± 0.4	44.9^f^	190,000	196,400	193,200	82,185	88,585	85,385	1.76	1.82	1.79
T2	NP_0_K + FYM + LPSBF	26.5 ± 0.6	28.4 ± 0.3	27.5^e^	46.4 ± 0.3	48.4 ± 0.1	47.4^e^	208,800	218,400	213,600	100,861	110,461	105,661	1.93	2.02	1.98
T3	NP_0_K + FYM + CCB	24.6 ± 0.2	25.1 ± 0.6	24.9^f^	46.0 ± 0.3	46.5 ± 0.1	46.2^ef^	202,000	206,000	204,000	93,885	97,885	95,885	1.87	1.91	1.89
T4	NP_75_K + FYM	39.2 ± 0.9	37.2 ± 0.7	38.2^c^	47.5 ± 0.8	51.5 ± 0.8	49.5^d^	241,200	250,400	245,800	130,520	139,720	135,120	2.18	2.26	2.22
T5	NP_75_K + FYM + LPSBF	42.3 ± 0.9	43.4 ± 0.7	42.9^b^	52.6 ± 0.7	53.8 ± 0.5	53.2^c^	246,800	254,400	250,600	135,996	143,596	139,796	2.23	2.30	2.26
T6	NP_75_K + FYM + CCB	40.1 ± 0.1	40.1 ± 0.8	40.1^c^	48.5 ± 0.5	52.6 ± 0.6	50.6^d^	244,600	251,800	248,200	133,620	140,820	137,220	2.20	2.27	2.24
T7	NP_100_K + FYM	43.2 ± 0.4	45.2 ± 0.3	44.2^b^	53.9 ± 0.8	56.8 ± 0.4	55.4^b^	277,200	284,400	280,800	165,565	172,765	169,165	2.48	2.55	2.52
T8	NP_100_K + FYM + LPSBF	46.1 ± 0.2	49.1 ± 0.7	47.6^a^	57.5 ± 0.4	58.9 ± 0.8	58.2^a^	316,400	335,200	325,800	204,641	223,441	214,041	2.83	3.00	2.92
T9	NP_100_K + FYM + CCB	45.8 ± 0.6	47.0 ± 0.8	46.4^a^	56.4 ± 0.5	58.2 ± 0.5	57.3^a^	300,800	307,200	304,000	188,865	195,265	192,065	2.69	2.74	2.72
T10	NP_100_K without FYM	27.1 ± 0.1	29.7 ± 0.2	28.4^e^	52.7 ± 0.3	53.9 ± 0.6	53.3^c^	238,000	248,000	243,000	126,365	136,365	131,365	2.13	2.22	2.18
T11	NP_100_K + LPSBF without FYM	29.5 ± 0.2	34.0 ± 0.8	31.8^d^	53.5 ± 0.5	54.7 ± 0.6	54.1^bc^	240,600	249,800	245,200	128,841	138,041	133,441	2.15	2.24	2.19
T12	NP_100_K + CCB without FYM	28.4 ± 0.1	32.5 ± 0.7	30.5^d^	53.1 ± 0.7	54.0 ± 0.3	53.6^bc^	239,000	248,200	243,600	127,065	136,265	131,665	2.14	2.22	2.18
Mean		34.6^B^	36.3^A^		51.1^B^	52.9^A^										

Replications: 3, Design: RBD, Plot size: 5 m × 4 m; Date of sowing: 20.10.2022 and 23.10.2023. Values represent the mean of three replicates with 10 plants each. Values with different letters in a column differ significantly from one another with two-way ANOVA post hoc Tukey's-b test at *p* ≤ 0.05.

pNP, p-nitrophenol; TPF, triphenyl formazan; CCB, charcoal-based consortium biofertilizer; LPSBF, liquid phosphate-solubilizing bacterial formulation.

Likewise, the shoot length observed with the treatment T8 was significantly higher than all other treatments which exhibited an increase of 10.0% in the shoot length over the control treatment T7 (NP_100_K + FYM). The shoot length observed with the treatment T5 (NP_75_K + FYM + LPSBF) was also statistically not different than that observed with the treatment T7 (NP_100_K + FYM). The shoot length though decreased in the treatments without FYM compared to the treatments with FYM, however, the shoot length observed with the treatment T11 (NP_100_K + LPSBF without FYM) was statistically not different from the control treatment T7 (NP_100_K + FYM). Similarly, the root length was also improved with the use of the bacterial formulations with the highest improvement exhibited by the treatment T8 recorded with an increase of 7.3% over the control treatment T7 (NP_100_K + FYM). Among the 2 years, the shoot and root length of potato observed in the year 2023–24 was significantly higher than the shoot length recorded in the year 2022–23.

The mean fresh and dry weight of 2 years also exhibited a significant improvement with the application of liquid PSB formulation along with different P doses compared to their respective uninoculated controls (Table 4). The highest fresh and dry weight recorded with the treatment T8 (NP_100_K + FYM + LPSBF) was significantly higher than all other treatments. The fresh weight observed with the treatment T9 (NP_100_K + FYM + CCB) was also significantly higher than the control treatment T7 (NP_100_K + FYM), though significantly lower than the T8 (NP_100_K + FYM + LPSBF) treatment. The increase in fresh and dry weight with the treatment T8 (NP_100_K + FYM + LPSBF) was 11.6 and 14.7%, respectively, over the control treatment T7 (NP_100_K + FYM). A significant decrease in plant fresh and dry weight was observed with the treatments without FYM when compared to treatments with FYM. The mean fresh as well as dry weight of potato recorded in 2023–24 was significantly higher than that observed in 2022–23.

Likewise, the chlorophyll content was also recorded to be highest with the treatment T8 (NP_100_K + FYM + LPSBF). However, it was not significantly different from treatments T5, T6, T7, and T9 (Table 5). The increase in chlorophyll content with the treatment T8 was found to be 3.9% over the treatment T7 (Table 5).

The normalized difference vegetation index (NDVI) of potato leaves also exhibited an improvement with the application of liquid PSB formulation though, it did not show much variation among the treatments (Table 5). The highest increase in NDVI observed with the treatment T8 (NP_100_K + FYM + LPSBF) was statistically similar to all other treatments except T1 (NP_0_K + FYM), T2 (NP_0_K + FYM + LPSBF) and T3 (NP_0_K + FYM + CCB). The average NDVI content in 2023–24 was slightly higher than in 2022–23, although the difference was not statistically significant.

#### 3.4.2 Yield parameters

The yield parameters were also improved significantly with the application of microbial formulations with the highest improvement recorded with the treatment T8 (NP_100_K + FYM + LPSBF; Tables 5, 6).

The mean number of tuber per plant were found to be the highest with the treatment T8 (NP_100_K + FYM + LPSBF), which was significantly higher than all the other treatments. An increase of 13.6% in tuber number was recorded with the treatment T8 over its respective control treatment T7 (NP_100_K + FYM). The tuber number decreased significantly in treatments without FYM as compared to their respective FYM-amended control treatments. Furthermore, the mean tuber number of 2 years recorded during 2023–24 was significantly higher than that observed in 2022–23 (Table 5).

Likewise, the liquid PSB formulation also significantly improved the mean tuber yield of 2 years compared to their corresponding uninoculated controls (Table 5). The highest tuber yield was recorded with the treatment T8 (NP_100_K + FYM + LPSBF), which was significantly higher than all other treatments but statistically not different than the treatment T9 (NP_100_K + FYM + CCB). An increase of 16.1% in mean tuber yield of 2 years was recorded with the treatment T8 over its control treatment T7. However, the tuber yield differed significantly between treatments without FYM and with FYM. The treatments T10, T11, and T12 (without FYM) showed significantly lower yields compared to the treatment T7 (NP_100_K + FYM). Furthermore, among the 2 years, the tuber yield observed in the year 2023–24 was significantly higher than the tuber yield obtained in the year 2022–23.

The mean haulm yield of potato of 2 years also exhibited improvement with the application of liquid PSB formulation with the highest haulm yield recorded with the treatment T8 (NP_100_K + FYM + LPSBF), which was significantly higher than all other treatments but statistically not different from the treatment T9 (NP_100_K + FYM + CCB). The increase in haulm yield with the treatment T8 was found to be 3.6% over its respective control treatment T7 (Table 5).

In different grade size category, the mean tuber yield of 2 years also showed improvement with the application of liquid PSB formulation compared to the uninoculated controls (Table 6). The highest yield in all the grade size categories was observed with the treatment T8 (NP_100_K + FYM + LPSBF), which was significantly higher than all other treatments but statistically not different from the treatment T9 (NP_100_K + FYM + CCB) in the grade size 25–35 mm. In the 45–55 mm grade size category, the yield observed with the treatment T8 (NP_100_K + FYM + LPSBF) was statistically not different from the tuber yield observed with treatments T5 (NP_75_K + FYM + LPSBF), T7 (NP_100_K + FYM) and T9 (NP_100_K + FYM + CCB). In the >55 mm grade size, the tuber yield observed with the treatment T8 (NP_100_K + FYM + LPSBF) was statistically similar to that tuber yield observed with the treatment T9 (NP_100_K + FYM + CCB). An increase in tuber yield of 8.4% in 25–35 mm grade, 3.9% in 45–55 mm grade and 12.3% in the grade >55 mm was observed with treatment T8 over the control treatment T7 (NP_100_K + FYM). The mean tuber yield with a grade size of 45–55 mm and >55 mm recorded in 2023–24 was significantly higher than that observed in 2022–23.

#### 3.4.3 Nutrient content of haulm and tuber

The mean N, P, and K contents of haulm and tuber of 2 years showed a significant improvement with the application of liquid PSB formulation with 0%, 75%, and 100% P doses compared to their respective uninoculated controls (Table 7). The highest N and P content of haulm was recorded with treatment T8 (NP_100_K + FYM + LPSBF), which was significantly higher than all other treatments. However, the K content observed with the treatment T8 though significantly higher than all other treatments was statistically similar with the treatment T9 (NP_100_K + FYM + CCB). An increase of 6.3 in N content, 18.2% in P content and 7.1% in K content of haulm was recorded with the treatment T8 over the control treatment T7.

Likewise, tuber N, P and K content also showed improvement with the application of PSB formulation with the highest improvement exhibited by the treatment T8 (NP_100_K + FYM + LPSBF). The nitrogen content in tubers observed with treatment T8 was significantly different from all treatments except for the treatment T9 (NP_100_K + FYM + CCB). However, the increase in tuber P and K content shown by the treatment T8 was significantly higher than all other treatments (Table 7). An increase of 5.7% in nitrogen content, 25.8% in P content and 11.2% in potassium content of tuber was observed with treatment T8 over the control treatment T7 (NP_100_K + FYM). However, the nutrient content of the haulm and tuber significantly decreased in treatments without FYM compared to their respective treatments with FYM.

#### 3.4.4 Soil parameters

A non-significant change in the soil pH was observed with the application of liquid PSB formulation with different doses of P over their respective uninoculated control treatments in both the years (Table 8). The pH values ranged from 7.39 to 7.45 during 2022–23 and from 7.83 to 7.94 in 2023–24.

The application of the liquid PSB formulation led to an increase in soil electrical conductivity (EC), though the increase was found to be non-significant (Table 8). The EC values ranged from 0.247 to 0.253 dS m^−1^ in 2022–23 and from 0.267 to 0.272 dS m^−1^ during 2023–24 with the highest soil EC recorded with the treatment T8 (NP_100_K + FYM + LPSBF) and T5 (NP_75_K + FYM + LPSBF) followed by T7 (NP_100_K + NP_100_K + FYM) and T11 (NP_100_K + LPSBF without FYM).

Likewise, the soil organic carbon (OC) showed slight improvement with the application of liquid PSB formulation with different doses of P compared to their respective uninoculated controls (Table 8). The organic carbon content ranged from 0.34 to 0.45% in 2022–23 and from 0.36 to 0.46% in 2023–24, with the highest soil OC recorded with the treatment T8 (NP_100_K + FYM + LPSBF). The organic content recorded in the treatments with FYM was significantly higher than the organic carbon observed in the treatments without FYM. The soil pH, EC and OC observed in the year 2023–24 was statistically not different from the OC observed in the year 2022–23.

The liquid PSB formulation also significantly affected the soil available N, P and K contents of 2 years (Table 8). The highest available nutrients were recorded with the treatment T8 (NP_100_K + FYM + LPSBF) with an increase of 1.85% in N, 11.35% in P and 1.45% in K content over the control treatment T7. The nutrient content, found in treatments without FYM was significantly lower than their respective treatments with FYM. Among the 2 years, the mean available N and K content of soil during 2023–24 was higher but not statistically different from that observed in the year 2022–23. However, the mean available P content in soil recorded during 2023–24 was significantly higher than that of 2022–23.

Additionally, the total microbial population of potato rhizosphere was positively influenced by the liquid PSB formulation during both the years, though the improvement observed was non-significant (Table 9). The initial bacterial population, recorded at 1.2 × 10^6^ and 5.6 × 10^6^ CFU ml^−1^, increased to 6.61 × 10^8^ and 7.24 × 10^8^ CFU ml^−1^ during the 2022–23 and 2023–24, respectively. Similarly, the fungal population rose from an initial count of 1.3 × 10^3^ and 2.3 × 10^3^ CFU ml^−1^ to 1.99 × 10^4^ and 2.45 × 10^4^ CFU ml^−1^ in 2022–23 and 2023–24, respectively. Likewise, the population of phosphate-solubilizing microorganisms also showed an increase from an initial count of 2.04 × 10^5^ and 2.09 × 10^5^ CFU ml^−1^ to 8.51 × 10^8^ and 8.91 × 10^8^ CFU ml^−1^ during 2022–23 and 2023–24, respectively. Similarly, actinomycetes populations increased from an initial value of 2.91 × 10^5^ and 2.90 × 10^5^ CFU ml^−1^ to 7.76 × 10^7^ and 7.24 × 10^7^ CFU ml^−1^ in 2022–23 and 2023–24, respectively. The diazotrophs population, initially 2.14 × 10^5^ and 2.18 × 10^5^ CFU ml^−1^, increased to 4.17 × 10^6^ and 5.25 × 10^6^ CFU ml^−1^during the two cropping years. Among treatments, the highest bacterial, fungal, phosphate-solubilizing, actinomycetes, and diazotrophs populations were recorded in the treatment T8 (NP_100_K + FYM + LPSBF), though increases were statistically similar with all other treatments.

The liquid PSB formulation also significantly affected the soil alkaline phosphatase and dehydrogenase activity of 2 years compared to the control treatments (Table 10). The highest enzyme activities were recorded with the treatment T8 (NP_100_K + FYM + LPSBF) which was significantly higher than all other treatments but statistically similar to the treatment T9 (NP_100_K + FYM + CCB). The treatment T8 exhibited an increase of 7.69% in alkaline phosphatase activity and 5.05% in soil dehydrogenase activity over the control T7. The enzyme activities significantly decreased with the treatments without FYM compared to their respective treatments with FYM. Among the 2 years, the mean alkaline phosphatase as well as dehydrogenase activity was significantly higher in the year 2023–24 than that observed in the year 2022–23.

The results indicated that the tuber inoculation with the liquid formulation of *Bl* PRPSB_10_ and *Pp* PRPSB_38_ increased the mean net returns by ₹44,876 ha^−1^ and mean B:C ratio by 0.40 in comparison to control treatment T7 (₹169,165 ha^−1^, 2.52, respectively) (Table 10).

## 4 Discussion

Potato (*Solanum tuberosum* L.) is one of the world's most important food crops, grown extensively for its high calorific value and versatility in human diets. Phosphorus is a vital macronutrient required for optimal tuber initiation, enlargement, and overall yield potential of potato crop. However, 98% of Indian soils and 36.4% of Punjab soils are deficient in available P due to its high fixation in insoluble forms by calcium in alkaline soils and by iron and aluminum in acidic soils (Sharma et al., 2016; Ibrahim et al., 2022). This results in low phosphorus use efficiency, with plants typically able to utilize only a small fraction of the applied phosphorus fertilizers. To address these challenges, the use of PSB has emerged as a promising eco-friendly strategy. Given the intensive nutrient demands and short crop cycle of potato, integrating PSB with chemical fertilizers and organic inputs like farmyard manure holds significant potential for improving growth, yield and phosphorus availability while sustaining soil health.

Therefore, in the present study, two strains *Bacillus licheniformis* and *Pseudomonas putida* exhibiting solubilization of three different phosphate substrates were utilized for the development of a liquid formulation for use in potato crop. In soil, phosphorus predominantly exists in insoluble forms such as tricalcium phosphate, iron phosphate, aluminum phosphate and rock phosphates. In general *Bacillus* and *Pseudomonas* spp. are well known phosphate solubilizers (Vyas and Gulati, 2009; Li et al., 2025; Kaur et al., 2025). *Bacillus licheniformis* and *Pseudomonas putida* were identified as efficient PSB strains, exhibiting a considerably higher phosphate solubilizing index compared to values reported in earlier studies (Karpagam and Nagalakshmi, 2014; Joshi et al., 2023; Rana et al., 2023). Quantitative estimation revealed that both strains efficiently solubilised three insoluble phosphates, with tricalcium phosphate being the most easily solubilized compared to rock phosphate and iron phosphate, as also reported in earlier studies (Gulati et al., 2008; Vyas and Gulati, 2009; Ateş, 2023).

Pathogenicity screening is a fundamental prerequisite while screening bacteria for biofertilizer development, as it enables the identification and elimination of potentially harmful bacterial strains. Moreover, such screening minimizes the risk of disseminating antibiotic resistance genes, thereby upholding biosafety standards and promoting environmental sustainability prior to field application (Tariq et al., 2022). Both the strains showed no haemolysis on blood agar, confirming the environmental safety of these strains to be employed as biofertilizers.

Additionally, testing compatibility between strains has also been widely used as a criterion for selecting strains during the development of inoculants (Vyas et al., 2025). The potential PSB strains *B. licheniformis* PRPSB_10_ and *P. putida* PRPSB_38_ showed uninterrupted growth at their intersections, suggesting their ability to coexist without antagonistic effects (Figure 2). This observation holds significance for their potential use as a dual culture in fields.

According to the Fertilizer Control Order (FCO) specifications (1985), PSB liquid formulations should maintain a minimum count of 1.0 × 10^8^ CFU ml^−1^. Formulation development using trehalose and carboxymethyl cellulose (CMC) significantly improved shelf-life, maintaining viable count above FCO standards for over 365 days (Figures 3, 4). Liquid formulations without additives showed rapid decline in cell viability, highlighting the importance of protectants for long-term storage (Aeron et al., 2012). Trehalose functions as a reserve carbohydrate and is mobilized under stress conditions such as desiccation, osmotic pressure, and temperature extremes and its role in enhancing the shelf life of liquid bioformulations is well-supported by earlier studies (Fillinger et al., 2001; Manimekalai and Kannahi, 2018; Chompa et al., 2024). Likewise, CMC is a cost-effective, non-ionic, water-soluble polymer known to act as an effective cell protectant even at low concentrations. Its moisture retention and viscosity-enhancing properties contribute to the stabilization of viable cells (Almeida et al., 2025; Hashem et al., 2025).

The present study demonstrated the efficacy of liquid phosphate-solubilizing bacterial (LPSBF) formulation containing *Bl*PRPSB_10_ and *Pp*PRPSB_38_ (LPSBF) in enhancing growth, yield, nutrient content and soil fertility of potato under field conditions. Field evaluation over 2 years revealed that LPSBF application, particularly with 100% NPK and FYM (T8), significantly improved the growth parameters, nutrient content, yield parameters and soil properties compared to the uninoculated control and the PAU recommended biofertilizer (CCB). Nutrient analysis showed increased N, P, and K content in shoots and tubers as well as soil available nutrients with LPSBF, reflecting improved nutrient mobilization and uptake through phosphate solubilization (Lin et al., 2023).

The application of liquid PSB formulation notably enhanced potato yield attributes, with treatment T8 recording the highest increases of 13.6% in tuber number, 16.2% in tuber yield, and 3.6% in haulm yield over the control (Tables 5, 6). These results corroborate earlier findings where PSB inoculation significantly improved tuber yield and tuber formation in potato through enhanced nutrient availability and root activity (Malboobi et al., 2009; Kaur et al., 2022). The higher haulm yield indicated vigorous vegetative growth and efficient photosynthetic activity, which contributes to better tuber development. Improved haulm yield also reflects enhanced nutrient uptake and overall plant health under favorable treatments (Chauhan et al., 2023). The microbial population in the potato rhizosphere was also increased with bacterial treatment, indicating successful colonization and activity of inoculated strains, supporting previous findings on PSB proliferation under field conditions (Stephen et al., 2015; Talwar et al., 2017). The increased soil dehydrogenase activity with the application of liquid formulation indicated enhanced microbial oxidation, reflecting greater microbial biomass and activity in the soil.

Additionally, the integration of the phosphate-solubilizing liquid formulation (LPSBF) with the farmyard manure and fertilizers at the recommended doses not only enhanced the potato productivity but also led to the economic benefits in terms of mean net returns of ₹44,876 ha^−1^ to the farmers.

## 5 Conclusion

Microbial formulations/biofertilizers are an indispensable part of the integrated nutrient management system. The carrier based formulations have a short shelf life and thus liquid formulations are considered superior due to prolonged shelf life, ease of handling and transportation. In the present study, a liquid formulation containing two phosphate-solubilizing bacterial strains was developed. The formulation with 0.1% CMC for *Bacillus licheniformis* and 5 mM trehalose for *Pseudomonas putida* effectively maintained bacterial viability for up to 1 year. Two-year field trials with the liquid formulation confirmed significant improvements in plant growth, nutrient uptake, yield (16.1% higher), and soil health, demonstrating the potential of the PSB formulation as an effective bioinoculant for improving potato productivity under Punjab's agro-climatic conditions. The average net returns gained by ₹44,876 ha^−1^ and the better cost-benefit ratio of 2.52 indicated that the integrated nutrient management strategy is economically sustainable and profitable for the farmers over the conventional methods that do not use bioinoculant applications.

## Data Availability

The datasets presented in this study can be found in online repositories. The names of the repository/repositories and accession number(s) can be found in the article/supplementary material.
